# The G protein modifier KCTD5 tunes the decoding of neuromodulatory signals necessary for motor function in striatal neurons

**DOI:** 10.1371/journal.pbio.3003117

**Published:** 2025-04-15

**Authors:** Douglas C. Sloan, Yini Liao, Forest Ray, Brian S. Muntean

**Affiliations:** Department of Pharmacology and Toxicology, Medical College of Georgia, Augusta University, Augusta, Georgia, United States of America; Rutgers Robert Wood Johnson Medical School, UNITED STATES OF AMERICA

## Abstract

G proteins (Gα and Gβγ subtypes) drive adenylyl cyclase type 5 (AC5) synthesis of cAMP in striatal neurons, which is essential for motor coordination. KCTD5 directly interacts with Gβγ to delimit signaling events, yet downstream impact of KCTD5 in striatal circuits is not known. Here, generation of a conditional *Kctd5* knockout mouse identified that loss of striatal KCTD5 leads to a dystonic phenotype, coordination deficits, and skewed transitions between behavioral syllables. 2-photon imaging of a cAMP biosensor revealed electrically evoked dopaminergic responses were significantly augmented in the absence of KCTD5 in striatal circuits. cAMP sensitization was rescued *in* *situ* by expression of a Gβγ-scavenging nanobody and motor deficits were partially rescued *in* *vivo* by pharmacological antagonism of the indirect striatal cAMP pathway. Therefore, KCTD5 acts as a brake on cAMP signaling in striatal neurons important for tuning dopaminergic signaling and motor coordination.

## Abstract 2

Dopamine modulates activity of striatal neurons via G proteins, but how this signaling is modulated by G protein modifiers *in* *vivo* is not clear. This behavioral and neuroimaging study shows *in* *vivo* that the G protein modifier KCTD5 regulates G proteins in the striatum and coordinates motor control via cAMP signaling.

## Introduction

Coordination of locomotor activity is guided by neuromodulators, such as dopamine and adenosine, that activate G protein-coupled receptors (GPCRs) [1,2]. One common downstream pathway is the synthesis of cAMP by adenylyl cyclase (AC) enzymes [3,4], where numerous players feed into the regulatory network of cAMP signal transduction [5]. Pathogenic mutations in many of these genes (such as *DRD2*, *GNAL*, *GNAO1*, *ADCY5*, and *PDE10A*) have been identified in patients with movement disorders (such as dystonia and dyskinesia) [6–11]. A striking number of such clinical variants are enriched in the striatum [12], which is a core component of the basal ganglia that enables motor control [13]. Striatal circuitry facilitates locomotion by two parallel outputs of striatal medium spiny neurons (MSNs): the direct pathway enriched in dopamine 1 receptor (D1-MSN or dMSN), and the indirect pathway enriched in dopamine 2 receptor (D2-MSN or iMSN) [14]. While understanding cAMP processing in striatal neurons has implications for the management of various movement disorders, the unraveled complexity of how signaling processes unfold in intact circuits remains unclear.

The dominant striatal cyclase (AC5) [15] is stimulated by Gαs/olf to generate cAMP, inhibited by Gαi, and conditionally sensitized by Gβγ release from Gαo [16,17]. Therefore G protein modifiers are well-positioned to adjust striatal cAMP and potential downstream impact on motor properties. Potassium channel tetramerization domain 5 (KCTD5) has recently been appreciated to bind Gβγ in an agonist-induced fashion [18,19]. We previously reported KCTD5 influences G protein-mediated cAMP signaling in cultured neurons [20]. This is thought to be the case based on reports that KCTD5 is a ubiquitin ligase adapter for Gβ1, which leads to degradation of Gβγ subunits [19,21–23] and plays a prominent role in AC5 sensitization in cultured cells [18,20,24]. However, sensitization of AC5 in striatal circuits has not been well studied and the role of KCTD5 in shaping neuromodulatory signal interrogation in intact circuits remains unknown. Nonetheless, the precedent to study KCTD5 mechanics further stems from observation that *Kctd5*^*+/-*^ mice exhibit irregularities in both motor coordination and learning [20]. However, it remains a mystery if behavioral deficits are specific to basal ganglia circuitry and striatal MSNs in particular.

To fill these gaps, we generated a conditional KCTD5 knockout mouse (*Kctd5 cKO*). We crossed *Kctd5 cKO* with established striatal Cre driver lines and a cAMP reporter mouse line for circuit-specific investigation of KCTD5. We report elimination of KCTD5 in dMSNs led to motor impairments associated with enhanced dopaminergic cAMP signaling while loss of KCTD5 in iMSNs promoted similar motor phenotypes with exacerbated dopaminergic and adenosinergic signaling. The magnified neuromodulatory responses were restored by scavenging Gβγ with a selective nanobody. Finally, behavioral deficits following genetic ablation of KCTD5 in iMSNs were partially rescued by pharmacologically antagonizing the adenosine A2A receptor. Our results collectively highlight the importance of KCTD5 in striatal physiology.

## Results

### Striatal KCTD5 knockout impairs motor coordination

**Fig 1:**
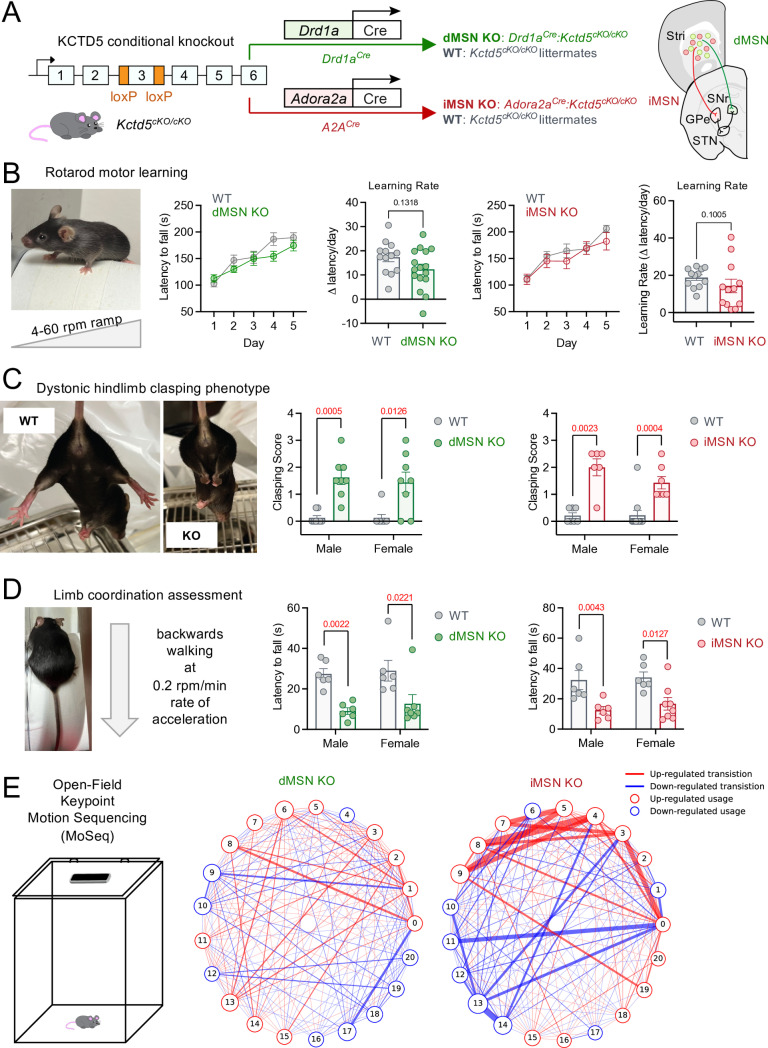
Striatal *Kctd5* knockout mice exhibit motor coordination deficits. **(A)** Scheme for striatal knockout selectively in dMSN or iMSN by crossing *Kctd5*^*cKO*^ with *Drd1a*^*Cre*^ or *Adora2a*^*Cre*^, respectively. **(B)** Accelerating rotarod latency to fall over five days with learning rate calculation (Day 1 latency subtracted from Day 5 latency, divided by number of days). dMSN KO (*n* = 16; 8 male, 8 female) compared with dMSN WT (*n* = 13; 5 male, 8 female) (nonparametric *t* test, Mann–Whitney test (*U* = 69), *p* = 0.1308). iMSN KO (*n* = 12; 5 male, 7 female) compared with iMSN WT (*n* = 12; 5 male, 7 female) (nonparametric *t* test, Mann–Whitney test (*U* = 43), *p* = 0.1005). **(C)** Hindlimb clasping score quantification. dMSN male, KO (*n* = 8) and WT (*n* = 8), nonparametric *t* test, Mann–Whitney test (*U* = 1), *p* = 0.0005. dMSN female, KO (*n* = 8) and WT (*n* = 8), nonparametric *t* test, Mann–Whitney test (*U* = 9.5), *p* = 0.0126. iMSN male, KO (*n* = 6) and WT (*n* = 7), nonparametric *t* test, Mann–Whitney test (*U* = 1.5), *p* = 0.0023. iMSN female, KO (*n* = 7) and WT (*n* = 11), nonparametric *t* test, Mann–Whitney test (*U* = 5.5), *p* = 0.0004. **(D)** Backwards walking latency to fall quantification. dMSN male, KO (*n* = 6) and WT (*n* = 6), nonparametric *t* test, Mann–Whitney test (*U* = 0), *p* = 0.0022. dMSN female, KO (*n* = 7) and WT (*n* = 6), nonparametric *t* test, Mann–Whitney test (*U* = 5), *p* = 0.0221. iMSN male, KO (*n* = 6) and WT (*n* = 6), nonparametric *t* test, Mann–Whitney test (*U* = 1), *p* = 0.0043. iMSN female, KO (*n* = 8) and WT (*n* = 6), nonparametric *t* test, Mann–Whitney test (*U* = 5), *p* = 0.0127. **(E)** Smartphone video recordings of mice in an open arena revealed behavioral syllable transition changes between dMSN (KO: *n* = 6, WT: *n* = 7) and iMSN (KO: *n* = 6 and WT: *n* = 6) cohorts. All data presented as mean ± SEM. The numerical data presented in this figure can be found in S1 Data. Additional data can be found in S1 and S2 Videos. The code related to Fig 1E is publicly available in a GitHub repository (https://github.com/BrianMunteanResearch/KCTD5_MoSeq_Analysis) and archived on Zenodo (https://doi.org/10.5281/zenodo.15019085).

We previously reported *Kctd5*^+/-^ mice, where haploinsufficiency significantly reduced KCTD5 protein level, exhibited profound deficits in motor function [20]. However, in our breeding colony, we were not able to obtain homozygous *Kctd5* knockout animals. Thus, in addition to circumventing embryonic lethality, we also wanted to understand the circuit-specific influence of KCTD5 elimination on motor performance. Therefore, we generated a *Kctd5* conditional knockout mouse (*Kctd5 cKO*) by insertion of loxP sites flanking exon 3, which is shared across each *Kctd5* transcript (ENSMUSG00000016946) (S1A Fig). Genomic sequencing identified founder mice, which we then backcrossed for five generations (S1B Fig). Verification of loxP insertion was readily monitored by standard PCR genotyping (S1C Fig). To validate Cre-mediated gene deletion, cultured primary striatal neurons (containing dorsal and ventral striata) from *Kctd5 cKO* were infected with AAV particles (Control: AAV-dTomato, or Cre: AAV-Cre-2A-dTomato) followed by western blot analysis (S1D Fig). KCTD5 protein was not detected in Cre-infected neurons compared with dTomato control (S1E Fig). Therefore, our *Kctd5* mouse model may serve as a useful tool for conditional elimination of KCTD5 protein. Accordingly, we crossed *Kctd5 cKO* with established striatal Cre driver lines for circuit-selective knockout of the direct (dMSN) and indirect (iMSN) pathways: dMSN KO (*Drd1a*^*Cre*^*:Kctd5*^*cKO/cKO*^) and iMSN KO (*Adora2A*^*Cre*^*:Kctd5*^*cKO/cKO*^) (**Fig 1A**). Mice were bred to obtain non-Cre homozygous *Kctd5 cKO* to serve as “wild-type (WT)” littermate controls and we evaluated motor performance between 2 and 4 months of age. Body weight measurements were similar between genetic crosses (S1F Fig). Equivalent numbers of each sex were utilized and we first noticed no differences in a measure of grip strength (S2A Fig). We next performed a detailed characterization of general ambulatory activity in an open-field arena (S2B–H Fig), which did not reveal anomalies between genotype or sex. Therefore, we moved to analyze motor activities sensitive to striatal impairments. Assessment on the accelerating rotarod revealed that KCTD5 is not required in either circuit for motor learning (**Fig 1B**). Voluntary movements were then measured by hindlimb motions in tail-suspended mice upon which we observed significant self-clasping in the absence of KCTD5 in either dMSNs or iMSNs (**Fig 1C**, S1 Fig, S2 Videos). Interestingly, the clasping phenotype is a rodent manifestation of dystonic movements observed in patients with hyperkinetic movement disorders [25]. Given the “dystonia-like” posture of these mice, we reasoned that limb coordination may be similarly compromised. Mice were challenged to coordinate motor sequences through reverse walking on a rotating beam. Indeed we recorded a significantly decreased motor ability in the absence of KCTD5 in either dMSNs or iMSNs (**Fig 1D**). To extract fine motor details with high precision, we applied Keypoint Motion Sequencing (MoSeq) analysis to unsupervised video recordings of mice during individual exploration in a large custom open field arena [26]. Keypoint MoSeq identified similar behavioral syllables in each cohort (S3A and S3B Fig). While dMSN KO exhibited nearly identical frequency of syllable usage compared with dMSN WT (S3C Fig), iMSN KO favored the usage of certain syllables over others compared with iMSN WT (S3D Fig). We therefore examined the relationship between incoming and outgoing syllables (S3E and S3F Fig). Our analysis revealed a striking genotype difference in transition rates between syllables (**Fig 1E**). Interestingly, iMSN KO revealed a more pronounced range of up- and down-regulated syllable transitions relative to dMSN KO. Our behavioral data thus establish that in striatal circuits KCTD5 is required for precision of motor coordination but may be dispensable for motor learning.

### Striatal KCTD5 knockout impairs dopaminergic signal decoding

**Fig 2:**
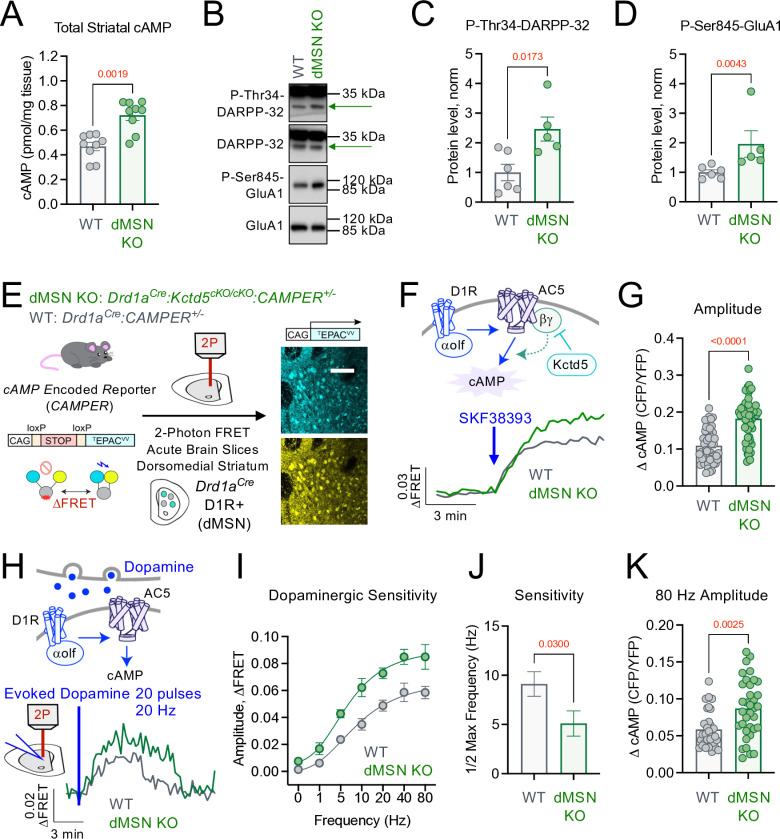
Loss of KCTD5 in dMSNs increases dopamine signaling downstream to cAMP. **(A)** ELISA quantification of cAMP in dorsal striatal brain punches. dMSN KO (*n* = 9) and WT (*n* = 9), nonparametric *t* test, Mann–Whitney *U* = 7, *p* = 0.0019. **(B)** Western blot from dMSN KO and WT striatal brain punches probing P-Thr34-DARPP32, DARPP32, P-Ser845-GluA1, and GluA1. Arrow indicates ~32 kDa band for DARPP32. **(C)** Quantification of P-Thr34-DARPP-32 (normalized to total DARPP-32). *n* = 6 (WT) and 5 (dMSN KO) mice, nonparametric *t* test, Mann–Whitney *U* = 2, *p* = 0.0173. **(D)** Quantification of P-Ser845-GluA1 (normalized to total GluA1). *n* = 6 (WT) and 5 (dMSN KO) mice, nonparametric *t* test, Mann–Whitney *U* = 0, *p* = 0.0043. **(E)** Scheme of 2-photon *CAMPER* imaging in dMSNs from acute brain slices (representative fluorescence from 300 μm acute slice). **(F)** Average trace of cAMP responses in dMSN to SKF38393 (10 μM), KO: *n* = 59 neurons/5 animals, WT: *n* = 53 neurons/5 animals. **(G)** Maximum SKF38393 (10 μM) induced cAMP amplitude in dMSN KO (59 neurons/5 animals) compared with dMSN WT (53 neurons/5 animals). Nonparametric *t* test, Mann–Whitney *U* = 483, *p* < 0.0001. **(H)** Average trace of evoked dopamine (20 Hz, 20 pulses) response in dMSN, KO: *n* = 31 neurons/5 animals, WT: *n* = 33 neurons/5 animals. **(I)** Evoked dopamine frequency-max cAMP amplitude response curve in dMSN KO and WT. *n* = 5 animals per genotype/frequency, *n* ≥ 24 neurons per frequency. **(J)** Quantification of half-max frequency of cAMP response obtained by curve four-parameter variable slope curve fitting [agonist] versus response from panel **F**. Unpaired parametric *t* test, *p* = 0.0300, F (1.174), DFn (26), Dfd (23). **(K)** Maximum cAMP amplitude to 80 Hz evoked response in dMSN KO (35 neurons/5 animals) compared with dMSN WT (30 neurons/5 animals). Nonparametric *t* test, Mann–Whitney *U* = 298, *p* = 0.0025. All data presented as mean ± SEM. The data underlying this figure can be found in S1 Raw Images. The numerical data presented in this figure can be found in S1 Data.

Dopaminergic neuromodulation of striatal neurons plays an essential role in movement [27,28]. Unlike fast ionotropic neurotransmission (e.g., glutamate), dopamine signals are transduced in striatal neurons through GPCRs that converge downstream to cAMP [5]. Therefore to understand how KCTD5 influences the striatal dopamine cascade in the physiological environment, we began by analyzing total cAMP content in dorsal striatal tissue punches. We observed a significant increase in baseline cAMP in dMSN KO compared with WT littermate controls upon ELISA-based quantification (**Fig 2A**). Striatal neurons can connect cAMP to downstream activity through PKA-mediated phosphorylation of Dopamine and cAMP-regulated phosphoprotein of 32 kDa (DARPP-32) at Thr34 (P-Thr34) and AMPAR subunit glutamate receptor 1 (GluA1) at Ser845 (P-Ser845) [29–32]. We probed total and phosphorylated levels of these two substrates with specific antibodies by western blot from striatal punches (**Fig 2B**). Consistent with elevated cAMP data, we observed a significant increase in both P-Thr34-DARPP-32 and P-Ser845-GluA1 in dMSN KO compared with WT littermates (**Fig 2C** and 2D). To next examine neuromodulatory signaling to cAMP, we crossed *Kctd5 cKO* with the *cAMP Encoded Reporter* (*CAMPER*) mouse line that conditionally expresses a FRET-based biosensor to monitor cAMP dynamics [33]. We then made acute brain slices to enable real-time 2-photon imaging of cAMP dynamics in D1-MSNs in intact circuits to compare dMSN KO (*Drd1a*^*Cre*^*:Kctd5*^*cKO/cKO*^*:CAMPER*^*+/-*^) with WT (*Drd1a*^*Cre*^*:CAMPER*^*+/-*^) (**Fig 2E**). Bath application of a D1R agonist (10 micromolar; SKF38393) induced a robust increase in cAMP that was significantly greater in dMSN KO compared with WT (Fig 2F and 2G), suggesting D1R signaling capacity is influenced by KCTD5.

We therefore wanted to investigate how endogenous dopamine signals were integrated downstream to cAMP. This was achieved by applying current through a stimulating electrode while recording concomitant cAMP transients in acute brain slices. Electrical stimulation (20 pulses at 20 Hz) in *Drd1a*^*Cre*^*:CAMPER*^*+/+*^ evoked a wave of cAMP similar to a previously reported optogenetic approach [33,34]. The same neurons were then stimulated 30 min later, which resulted in a roughly equivalent response (S4A Fig). We verified our approach by performing the same experiment with the inclusion of a D1R antagonist (SCH23390; 10 micromolar) during the second stimulation, which abolished the cAMP response (S4B Fig). To mimic tonic (~5 Hz) and phasic (>15 Hz) firing patterns of dopamine release [35], we applied varying frequencies of current while maintaining the same number of pulses (Fig 2H and 2I and S4C Fig). Interestingly, loss of KCTD5 significantly increased the sensitivity to dopamine as evidenced by a lower frequency required to generate half of the maximum response (**Fig 2J**). At the same time, the maximum amplitude was also significantly greater in dMSN KO compared with WT (**Fig 2K**).

**Fig 3:**
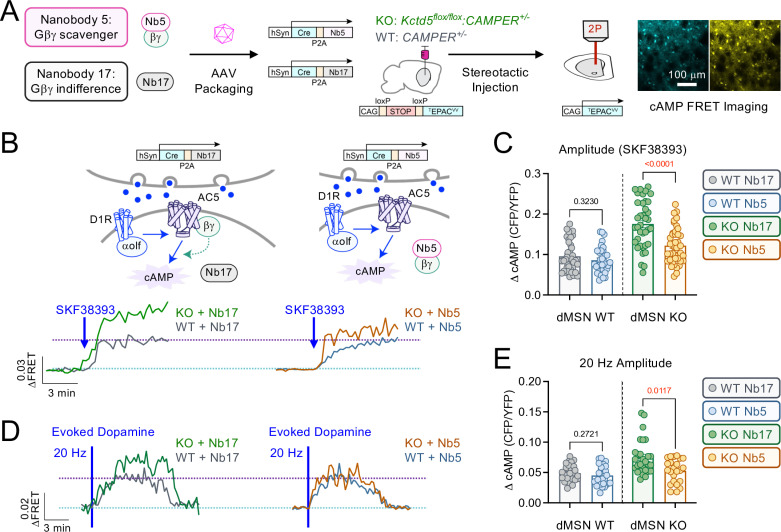
G βγ-selective nanobody restores dopamine efficacy in D1-MSN KCTD5 KO. **(A)** Nanobody expression via stereotaxic AAV injection in *CAMPER* mice for 2-photon imaging in acute brain slices (representative fluorescence from 300 μm acute slice). **(B)** Average trace of cAMP responses to SKF38393 (10 μM) in nanobody-expressing dMSN: KO-Nb17 (*n* = 38 neurons/8 animals), WT-Nb17 (40 neurons/9 animals), KO-Nb5 (*n* = 42 neurons/8 animals), WT-Nb5 (34 neurons/7 animals). **(C)** Maximum cAMP response to SKF38393 (10 μM) in nanobody-expressing dMSN: WT-Nb17 compared with WT-Nb5: Nonparametric *t* test, Mann–Whitney *U* = 588, *p* = 0.3230. KO-Nb17 compared with KO-Nb5: Nonparametric *t* test, Mann–Whitney *U* = 374, *p* < 0.0001. **(D)** Average trace of evoked dopamine (20 Hz, 20 pulses) response in nanobody-expressing dMSN: KO-Nb17 (*n* = 28 neurons/8 animals), WT-Nb17 (26 neurons/9 animals), KO-Nb5 (*n* = 23 neurons/8 animals), WT-Nb5 (28 neurons/7 animals). **(E)** Maximum cAMP response at 20 Hz in nanobody-expressing dMSN: WT-Nb17 compared with WT-Nb5: Nonparametric *t* test, Mann–Whitney *U* = 300, *p* = 0.2721. KO-Nb17 compared with KO-Nb5: Nonparametric *t* test, Mann–Whitney *U* = 190, *p* = 0.0017. All data presented as mean ± SEM. The numerical data presented in this figure can be found in S1 Data.

We, therefore, hypothesized that sensitization of the cAMP system in these neurons may be attributed to Gβγ. Our reasoning is 2-fold: (i) AC5 is the major striatal cyclase isoform [15] and its enzymatic activity following Gαs/olf stimulation is greatly increased in the presence of Gβγ [16], (ii) KCTD5 has been reported to bind Gβγ [19,21,22] thereby reducing cyclase sensitization [18,24]. Thus, our data suggest loss of KCTD5 enables Gβγ sensitization of D1R→AC5 response to synaptic dopamine. To test this hypothesis we expressed a genetically encoded nanobody scavenger of Gβγ (Nb5) [36] in the dorsal striatum via stereotaxic injection of AAV particles (AAV-hSyn-Cre-P2A-Nb5) (**Fig 3A**). A nanobody (Nb17) that does not interact with Gβγ was utilized as a control (AAV-hSyn-Cre-P2A-Nb17). In this strategy, AAV particles encode both Cre and the nanobody to take advantage of our conditional mouse models. Knockdown of KCTD5 was achieved with the *Kctd5*^*cKO/cKO*^*:CAMPER*^*+/-*^ strain, whereas *CAMPER*^*+/-*^ served as WT control. We then imaged cAMP responses to a saturating concentration of D1R agonist (10 micromolar; SKF38393) (**Fig 3B**). We observed that WT dMSNs elicited similar cAMP responses whether expressing Nb5 or Nb17 (**Fig 3C**), suggesting the approach did not influence intrinsic signaling properties. Strikingly, Nb5-expressing dMSN KO exhibited a significantly reduced cAMP response compared with Nb17. We next examined cAMP responses to evoked dopamine at 20 Hz, which represents phasic firing patterns of dopaminergic neurons. dMSN were identified based on directionality of cAMP response (i.e., neurons that increased cAMP in response to stimulation). Our results revealed that elevated cAMP response sensitivity and maximum signaling amplitude were partially rescued by nanobody-mediated inhibition of Gβγ (Fig 3D and 3E). These data support the notion that in the absence of KCTD5, there is greater availability of Gβγ to sensitize striatal AC activity and that scavenging Gβγ was able to rescue the enhanced cAMP signaling profile *in* *situ*.

**Fig 4:**
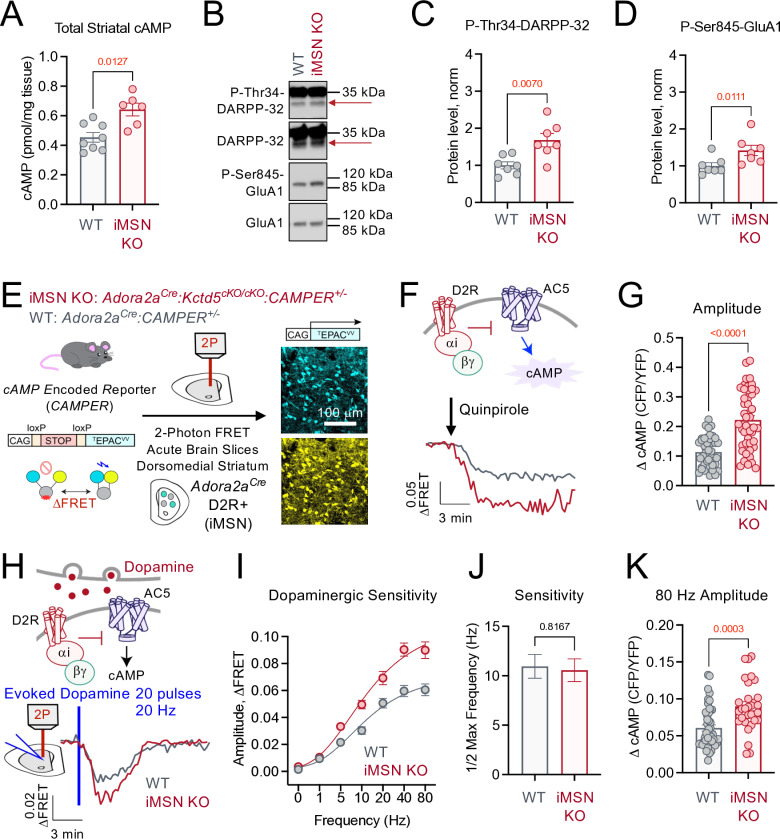
iMSN KO of KCTD5 increases dopamine cAMP signaling. **(A)** ELISA quantification of cAMP in dorsal striatal brain punches. iMSN KO (*n* = 6) and WT (*n* = 8), nonparametric *t* test, Mann–Whitney *U* = 5, *p* = 0.0127. **(B)** Western blot from iMSN KO and WT striatal brain punches probing P-Thr34-DARPP32, DARPP32, P-Ser845-GluA1, and GluA1. Arrow indicates ~32 kDa band for DARPP32. **(C)** Quantification of P-Thr34-DARPP-32 (normalized to total DARPP-32). *n* = 7 (WT) and 7 (iMSN KO) mice, nonparametric *t* test, Mann–Whitney *U* = 4, *p* = 0.0070. **(D)** Quantification of P-Ser845-GluA1 (normalized to total GluA1). *n* = 7 (WT) and 7 (iMSN KO) mice, nonparametric *t* test, Mann–Whitney *U* = 5, *p* = 0.0111. **(E)** Scheme of 2-photon *CAMPER* imaging in iMSNs from acute brain slices (representative fluorescence from 300 μm acute slice). **(F)** Average trace of cAMP responses in iMSN to Quinpirole (10 μM), KO: *n* = 47 neurons/6 animals, WT: *n* = 42 neurons/5 animals. **(G)** Maximum Quinpirole (10 μM) induced cAMP amplitude in iMSN KO (47 neurons/6 animals) compared with iMSN WT (42 neurons/5 animals). Nonparametric *t* test, Mann–Whitney *U* = 373, *p* < 0.0001. **(H)** Average trace of evoked dopamine (20 Hz, 20 pulses) response in iMSN, KO: *n* = 26 neurons/6 animals, WT: *n* = 38 neurons/5 animals. **(I)** Evoked dopamine frequency-max cAMP amplitude response curve in iMSN KO (6 animals, ≥26 neurons per frequency) and WT (5 animals, ≥29 neurons per frequency). **(J)** Quantification of half-max frequency of cAMP response obtained by curve four-parameter variable slope curve fitting [agonist] versus response from panel **F**. Unpaired parametric *t* test, *p* = 0.8167, F (1.220), DFn (28), Dfd (25). **(K)** Maximum cAMP amplitude to 80 Hz evoked response in iMSN KO (30 neurons/6 animals) compared with iMSN WT (44 neurons/5 animals). Nonparametric *t* test, Mann–Whitney *U* = 339, *p* = 0.0003. All data presented as mean ± SEM. The data underlying this figure can be found in S1 Raw Images. The numerical data presented in this figure can be found in S1 Data.

We next examined if neuromodulation in D2-MSN was impacted by loss of KCTD5. Here, baseline cAMP level was also significantly increased in dorsal striatal tissue punches in iMSN KO compared with WT (**Fig 4A**). Downstream cAMP/PKA targets, DARPP-32 and GluA1, were examined by western blot of striatal punches (**Fig 4B**). Analysis established that P-Thr34-DARPP32 and P-Ser845-GluA1 were both significantly increased in iMSN KO compared with WT (Fig 4C and 4D). As iMSNs are enriched with the D2R, which couples to Gαi for AC5 inhibition, we wondered if perhaps this signaling modality may contribute toward cAMP adjustments. Therefore, we utilized our *CAMPER* platform to compare D2R responses in brain slices from WT (*Adora2a*^*Cre*^*:CAMPER*^*+/-*^) and iMSN KO (*Adora2a*^*Cre*^*:Kctd5*^*cKO/cKO*^*:CAMPER*^*+/-*^) (**Fig 4E**). Interestingly, bath application of a D2R agonist (quinpirole; 10 micromolar) generated robust inhibition of cAMP that was significantly greater in iMSN KO compared with WT (Fig 4F and 4G). To examine synaptic dopamine decoding in these neurons, we utilized electrical stimulation to evoke cAMP responses. Electrical stimulation (20 pulses at 20 Hz) in *Adora2a*^*Cre*^*:CAMPER*^*+/-*^ induced robust cAMP inhibition that could be reproduced at a 30-minute interval and blocked by a D2R antagonist (sulpiride; 1 micromolar) (S5A and S5B Fig). We therefore established the stimulation frequency-cAMP response curve in WT and iMSN KO (Fig 4H and 4I; S5C Fig). Interestingly, KCTD5 did not influence evoked dopamine sensitivity (**Fig 4J**). Rather, loss of KCTD5 significantly increased cAMP response efficacy (**Fig 4K**). These data are at odds with the elevated basal cAMP that was observed in iMSN KO (**Fig 4**A). This led us to speculate that Gβγ sensitization of AC5 is likely a general feature of both MSN populations whereby increased basal cAMP in iMSNs enables larger cAMP clearance by PDE following D2R signaling.

**Fig 5:**
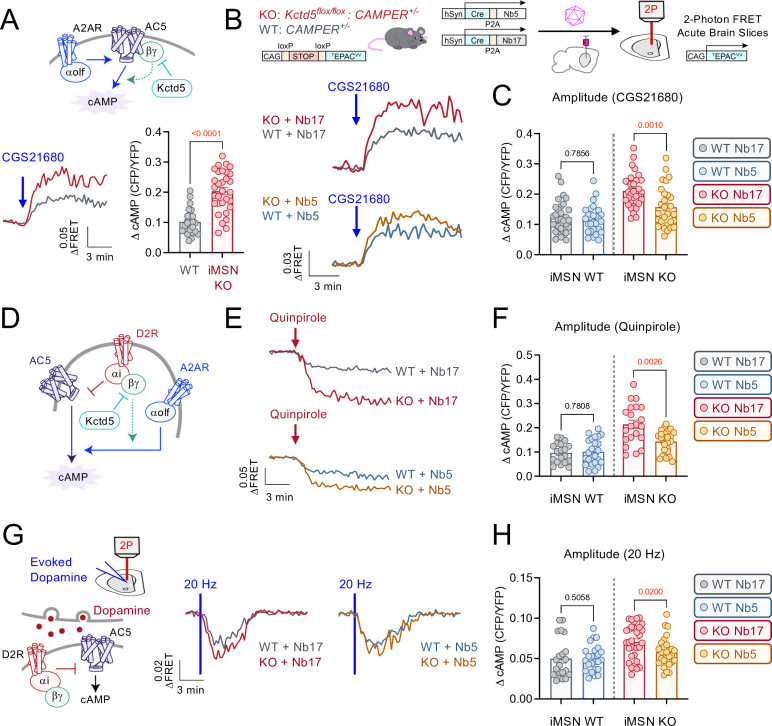
Examining impact of G **βγ** **sensitization on cAMP production in iMSN KCTD5 knockout.** **(A)** Maximum CGS21680 (10 μM) induced cAMP amplitude in iMSN KO (31 neurons/6 animals) compared with iMSN WT (25 neurons/5 animals). Nonparametric *t* test, Mann–Whitney *U* = 89, *p* < 0.0001. **(B)** Nanobody expression via stereotaxic AAV injection in *CAMPER* mice for 2-photon imaging in acute brain slices (representative fluorescence from 300 μm acute slice). Average trace of cAMP responses to CGS21680 (10 μM) in nanobody-expressing iMSN: KO-Nb17 (*n* = 32 neurons/8 animals), WT-Nb17 (32 neurons/9 animals), KO-Nb5 (*n* = 29 neurons/8 animals), WT-Nb5 (28 neurons/7 animals). **(C)** Maximum cAMP response to CGS21680 (10 μM) in nanobody-expressing iMSN: WT-Nb17 compared with WT-Nb5: Nonparametric *t* test, Mann–Whitney *U* = 429, *p* = 0.7856. KO-Nb17 compared with KO-Nb5: Nonparametric *t* test, Mann–Whitney *U* = 240, *p* = 0.0010. **(D)** Schematic of interplay between D2R and A2AR signaling in iMSN. **(E)** Average trace of cAMP responses to Quinpirole (10 μM) in nanobody-expressing iMSN: KO-Nb17 (*n* = 21 neurons/8 animals), WT-Nb17 (19 neurons/9 animals), KO-Nb5 (*n* = 24 neurons/8 animals), WT-Nb5 (24 neurons/7 animals). **(F)** Maximum cAMP response to Quinpirole (10 μM) in nanobody-expressing iMSN: WT-Nb17 compared with WT-Nb5: Nonparametric *t* test, Mann–Whitney *U* = 216, *p* = 0.7808. KO-Nb17 compared with KO-Nb5: Nonparametric *t* test, Mann–Whitney *U* = 122, *p* = 0.0026. **(G)** Average trace of evoked dopamine (20 Hz, 20 pulses) response in nanobody-expressing iMSN: KO-Nb17 (*n* = 31 neurons/8 animals), WT-Nb17 (21 neurons/9 animals), KO-Nb5 (*n* = 28 neurons/8 animals), WT-Nb5 (24 neurons/7 animals). **(H).** Maximum cAMP response at 20 Hz in nanobody-expressing iMSN: WT-Nb17 compared with WT-Nb5: Nonparametric *t* test, Mann–Whitney *U* = 222, *p* = 0.5058. KO-Nb17 compared with KO-Nb5: Nonparametric *t* test, Mann–Whitney *U* = 281.5, *p* = 0.0200. All data presented as mean ± SEM. The numerical data presented in this figure can be found in S1 Data.

One attractive candidate for supplying cAMP tone is the adenosine 2A receptor (A2AR), which is selectively expressed in iMSNs and couples to Gαs/olf [37,38]. Stimulation of iMSNs with a synthetic A2AR agonist (CGS21680; 10 micromolar) robustly increased cAMP and the response was significantly greater in iMSN KO compared with WT (**Fig 5A**). As the results aligned with our hypothesis of Gβγ sensitization, we applied our nanobody strategy in an attempt to curb excess signaling (**Fig 5B**). Indeed, expression of the Gβγ-scavenger Nb5 significantly reduced cAMP amplitude following A2AR stimulation (CGS21680; 10 micromolar) in iMSN KO whereas the Gβγ-indifferent Nb17 elicited no effect (**Fig 5C**). As sequestering Gβγ was sufficient to correct A2AR-mediated tone, our model predicted that D2R signaling in iMSN would also be rescued (**Fig 5D**). To test this hypothesis, we stimulated Nb-expressing iMSNs with the D2R agonist (quinpirole; 10 micromolar) and recorded cAMP responses (**Fig 5E**). We observed iMSN KO cAMP responses were significantly reduced in Nb5 neurons compared with Nb17 whereas the nanobodies did not have an impact on WT neurons (**Fig 5F**). To gauge the physiological inputs, we next recorded evoked dopamine responses in Nb-expressing neurons (**Fig 5G**). iMSN were identified based on directionality of cAMP response (i.e., neurons that decreased cAMP in response to stimulation). Similarly, synaptic D2R responses were significantly reduced in Nb5-expressing iMSNs compared with Nb17 (**Fig 5H**). Collectively, these data demonstrate iMSN cAMP tone is greatly increased in the absence of KCTD5 through Gβγ sensitization of AC, which results in enhanced dopaminergic and adenosinergic signal transduction.

### A2AR antagonism, but not D1R antagonism, rescues motor deficits in Kctd5 KO

**Fig 6:**
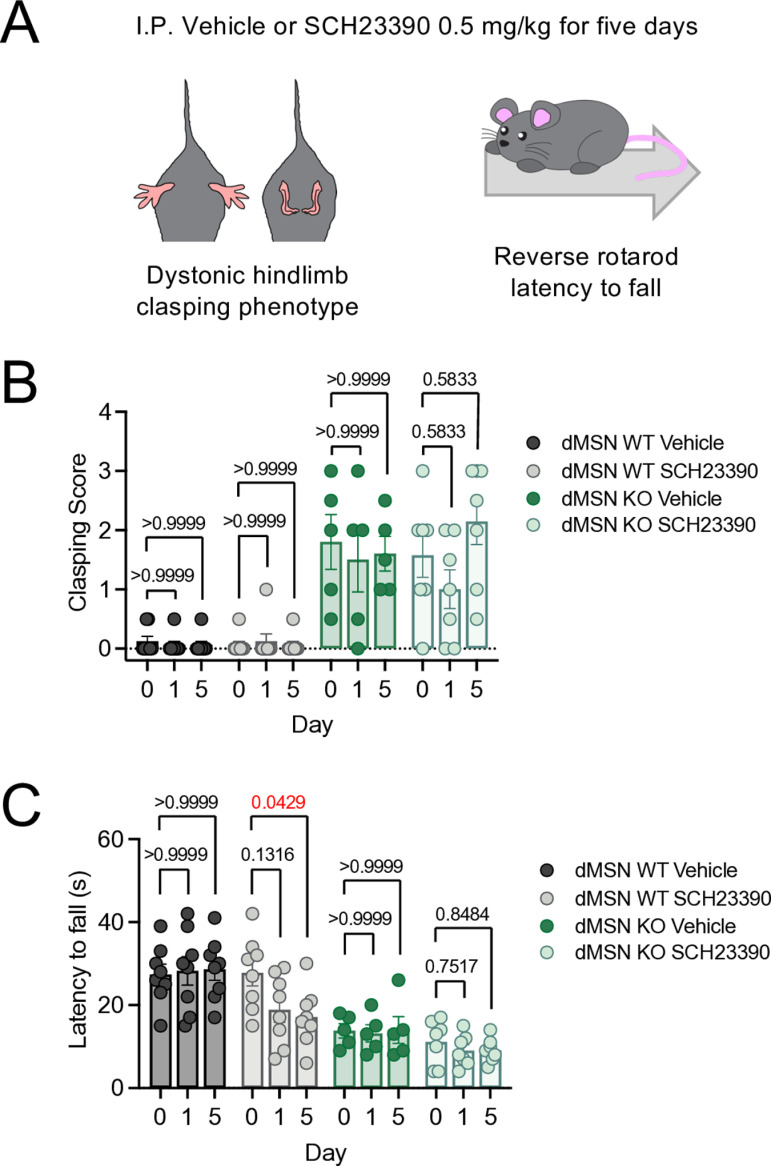
D1R antagonism does not rescue motor deficits in dMSN KCTD5 KO. **(A)** Schematic of mouse behavior assessment. **(B)** Quantification of hindlimb clasping at baseline (Day 0), acute (Day 1; single dose), and chronic (Day 5; daily injection for 5 days) following Vehicle or SCH23390 (0.5 mg/kg) in dMSN WT or dMSN KO. Behavior testing performed 30 min following I.P. injection. dMSN WT; *n* = 8 (vehicle), 8 (SCH23390). dMSN KO; *n* = 5 (vehicle), 7 (SCH23390). **(C)** Quantification of latency to fall during reverse rotarod. dMSN WT; *n* = 8 (vehicle), 8 (SCH23390). dMSN KO; *n* = 5 (vehicle), 7 (SCH23390). All data presented as mean ± SEM. The numerical data presented in this figure can be found in S1 Data.

**Fig 7:**
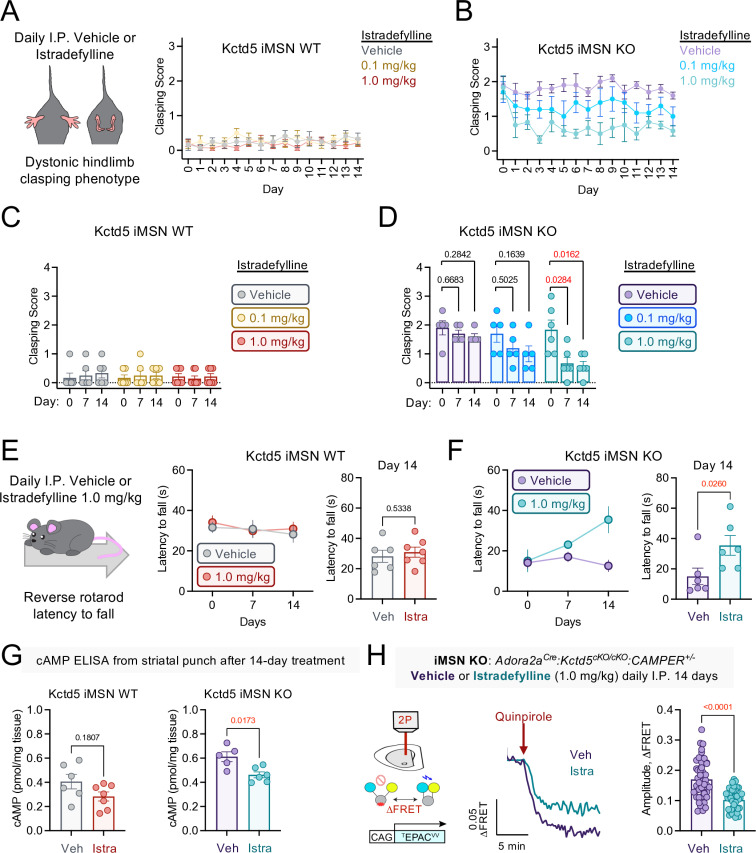
A2AR antagonism rescues behavioral and signaling deficits in iMSN KCTD5 KO. **(A)** Hindlimb clasping score following daily Istradefylline I.P. injections in iMSN WT; *n* = 6 (vehicle), 6 (0.1 mg/kg), 7 (1.0 mg/kg). **(B)** Hindlimb clasping score following daily Istradefylline I.P. injections in iMSN KO; *n* = 5 (vehicle), 5 (0.1 mg/kg), 6 (1.0 mg/kg). **(C)** Average clasping score for iMSN WT at Day 0, Day 7, and Day 14. **(D)** Average clasping score for iMSN KO at Day 0, Day 7, and Day 14. **(E)** iMSN WT latency to fall during reverse rotarod at Day 0, Day 7, and Day 14; *n* = 6 (vehicle), 7 (1.0 mg/kg). Comparison at Day 14: Nonparametric *t* test, Mann–Whitney *U* = 16, *p* = 0.5338. **(F)** iMSN KO latency to fall during reverse rotarod at Day 0, Day 7, and Day 14; *n* = 5 (vehicle), 6 (1.0 mg/kg). Comparison at Day 14: Nonparametric *t* test, Mann–Whitney *U* = 4, *p* = 0.0260. **(G)** ELISA quantification of cAMP in dorsal striatal brain punches. iMSN WT Vehicle (*n* = 6) and Istradefylline (*n* = 7), nonparametric *t* test, Mann–Whitney *U* = 11, *p* = 0.1807. iMSN KO Vehicle (*n* = 5) and Istradefylline (*n* = 6), nonparametric *t* test, Mann–Whitney *U* = 2, *p* = 0.0173. **(H).** Average trace of cAMP responses to Quinpirole (10 μM) in iMSN brain slices from 14-day treated iMSN KO: Vehicle (*n* = 47 neurons/6 animals), Istradefylline (40 neurons/7 animals). Comparison of maximum cAMP amplitude to Quinpirole (10 μM): nonparametric *t* test, Mann–Whitney *U* = 341.5, *p* < 0.0001. All data presented as mean ± SEM. The numerical data presented in this figure can be found in S1 Data.

To test our data *in* *vivo*, we examined if D1R antagonism would restore behavioral phenotypes in dMSN KO (*Drd1a*^*Cre*^*:Kctd5*^*cKO/cKO*^) and WT (*Kctd5*^*cKO/cKO*^) littermates. The D1R antagonist SCH23390 has been reported to impact motor coordination in rodents following a single intraperitoneal (I.P.) dose ranging from 0.1 to 1.0 mg/kg [39,40]. We established cohorts of naive mice to assess baseline clasping scores and latency to fall off the reverse rotarod (**Fig 6A**). The following day, SCH23390 (0.5 mg/kg) or saline was I.P. injected for clasping assessment and reverse rotarod 30 min later. We continued daily I.P. injections for five total days and then recorded both behaviors following the final administration. dMSN KO consistently displayed the clasping phenotype, which was absent in WT littermates. However, SCH23390 treatment did not impact clasping scores in either dMSN KO or WT (**Fig 6B**). Similarly, SCH22390 did not impact reverse rotarod performance in dMSN KO (**Fig 6C**). On the other hand, chronic SC23390 (Day 5) significantly reduced WT latency compared with baseline. While motor skill movement is indeed dependent on striatal D1R [41], the effect of SCH23390 in WT and not dMSN KO may be due to D1R expression in other brain centers. Notably, local motor cortex inhibition of D1R is sufficient to impede motor skills in mice [42,43]. Neuronal A2AR expression is relatively enriched in striatal iMSN [44], so we examined how an A2AR inhibitor would impact motor performance in iMSN KO (*Adora2a*^*Cre*^*:Kctd5*^*cKO/cKO*^) and WT (*Kctd5*^*cKO/cKO*^) littermates. We chose istradefylline as it has been approved for adjunctive therapy in Parkinson’s disease [45]. We first recorded baseline motor performance on day 0 (hindlimb clasping and reverse rotarod). Mice were given daily I.P. injections for 14 days (vehicle, 0.1 mg/kg, 1.0 mg/kg) followed by clasping assessment one hour after treatment (**Fig 7A** and **7B**). WT mice did not display clasping deficits at baseline or in response to IP injections (**Fig 7C**). iMSN KO exhibited clasping that was unchanged in response to vehicle whereas istradefylline induced a dose-dependent phenotype reduction (**Fig 7D**). We also examined backward walking in response to istradefylline by performing reverse rotarod at 7 and 14 days post-treatment. WT did not reveal differences in latency between vehicle and istradefylline (**Fig 7E**). Strikingly iMSN KO significantly improved motor performance on day 14 of istradefylline, but not day 7, with latency matching WT level (**Fig 7F**). Our model suggests A2AR blockade would reduce basal cAMP and concomitant D2R-mediated signaling. Therefore we measured total cAMP in dorsal striatal tissue punches following the 14-day treatment regimen (vehicle or 1.0 mg/kg). Istradefylline slightly reduced cAMP in WT mice, however the trend was not significant (**Fig 7G**). On the other hand, istradefylline significantly reduced total cAMP level in iMSN KO compared with vehicle treatment (**Fig 7G**). Finally, we treated *Adora2a*^*Cre*^*:Kctd5*^*cKO/cKO*^*:CAMPER*^*+/-*^ mice with either vehicle or istradefylline (1.0 mg/kg) for 14 days followed by 2-photon imaging in acute brain slices. Bath application of a D2R agonist (quinpirole; 10 micromolar) robustly inhibited cAMP and the response was significantly reduced in chronic istradefylline-treated mice (**Fig 7H**). Altogether, loss of KCTD5 in iMSNs leads to elevated cAMP signaling that can be rescued by A2AR inhibition. Moreover, behavioral deficits from loss of KCTD5 in iMSNs are also partially restored by an A2AR antagonist.

## Discussion

In this study, we developed a conditional KCTD5 knockout mouse that was utilized to uncover two major findings. First, loss of KCTD5 selectively in either D1- or D2-MSN leads to motor deficits including a dystonic phenotype. Second, we establish KCTD5 regulates Gβγ sensitization of AC5 to facilitate neuromodulatory signal decoding in striatal neurons. Additionally, our results support the mechanism of action for the A2AR antagonist istradefylline to rescue motor phenotypes and cAMP alterations in KCTD5 KO.

A combination of motor behavioral assessments revealed that loss of KCTD5 selectively in striatal circuits led to deficiencies in motor coordination but not motor learning. Motor learning deficits have been reported in *Adcy5* KO mice [46] as well as genetic models that indirectly reduce AC5 level (e.g., *Nf1* KO, *Pdcl* KO, *Kctd1* knockdown) [47–49]. We believe the absence of motor learning in our KO models supports the notion that KCTD5 likely exerts a more discrete role in tuning signal transduction. The dystonic epochs and coordination deficits observed in KCTD5 KO reflect hyperkinetic movement disorder pathology observed in patients with polymorphisms in signal transduction machinery, including *DRD2*, *GNAL*, *GNAO1*, and *GNB1* [6–9]. Strikingly, we observed overlap in phenotypes between KCTD5 deletion in either dMSN or iMSN. However, this aligns with deficits previously reported in *Kctd5*^*+/-* 20^, which models haploinsufficiency in both circuits. Curiously, we did not find sex differences between genotypes in any behavioral assays, which may be worth pointing out considering female patients may be overrepresented in a number of movement disorders [50,51].

Application of machine learning (Keypoint-MoSeq) identified discrepancies in behavioral syllable transitions but not overall usage between KCTD5 KO with WT littermates. Intriguingly, differences in transitions were notably more exaggerated following loss of KCTD5 in iMSN relative to dMSN. The greater severity in iMSN KO supports studies suggesting that motor irregularities (i.e., dystonic manifestations and chorea storms) disseminate from iMSN circuitry [52] as well as electrophysiological alterations in dystonia models [53]. Moreover, iMSNs project to the external Global Pallidus [14], which is often targeted for deep brain stimulation therapy in dystonia [54]. iMSNs, contrary to dMSNs, have also been identified to promote action abortion and switching between motor programs [55,56], thus supporting a greater disparity in syllable transition in iMSN KO relative to dMSN KO. Combined with our cAMP data, we were prompted to attempt to rescue behavioral profiles via antagonism at the A2AR with istradefylline, which is FDA approved for certain movement disorders [45]. Consistent with the role of striatal A2AR in movement [57], we found chronic istradefylline sufficient to rescue dystonic hindlimb clasping (partially) and coordinated backward walking (completely) relative to control. Notably, D1R antagonism was unable to rescue motor deficits in dMSN KO. We attribute these observations to widespread neuronal expression of D1R that presumably alters several nodes of circuitry [58,59]. Therfore, our study further highlights importance of striatal pharmacology in motor function, and istradefylline at the A2AR in particular.

We also report the influence of Gβγ-sensitization on striatal AC activity *in* *situ*, which is prominently shaped by KCTD5. Specifically, absence of KCTD5 in dMSN enhances D1R→cAMP whereas KCTD5 deletion in iMSN exacerbates A2AR→cAMP. Thus, our data support a model where KCTD5 acts as a brake on cAMP signaling by reducing Gβγ-sensitization of AC5. It has previously been reported that sequestering Gβγ from WT striatal membranes (via Grk2i peptide) significantly reduces AC activity [17] and submicromolar amounts of Gβγ sufficiently double AC5 catalytic activity [60]. AC5 regulation may be even more nuanced as a recent structure by Yen and colleagues suggests AC5 exists in an autoinhibited dimer where Gβγ may drive AC5 toward the active monomeric state [60]. Thus KCTD5, with its comparatively low affinity for Gβγ [22,24], is subtly poised to fine-tune signaling at AC5. Therefore during loss of KCTD5, AC5 is subject to a greater level of Gβγ-sensitization, as evidenced by our nanobody rescue experiments. Physiologically, blocking cyclase sensitization via the AC5 neddylation inhibitor MLN4924 reduces locomotor impact following alcohol and allodynia in an inflammatory pain model [61]. Additionally, cAMP sensitization is a prominently hypothesized mechanism of action for re-wiring striatal addiction circuits by drugs of abuse [34,62–65].

Thus, sensitization of AC5 is strongly connected to physiology, which leads to the question of what is the source of Gβγ that enables KCTD5 to play a role in cAMP sensitization in striatal neurons? Strikingly, we report similar cAMP sensitization in both dMSN and iMSN which suggests that KCTD5-Gβγ interplay is not receptor specific. Unlike certain KCTD (8, 12, 16) that interact with GABA_B_ receptors for membrane localization [66,67], KCTD5 in cell culture has been localized in the cytosol [23]. However, extensive studies in neurons have not yet been performed and therefore at this stage, we cannot rule out scaffolding to subcellular compartments. Nonetheless, our data support a receptor ambiguous mode for KCTD5 interaction with Gβγ, which matches existing literature regarding the agonist-induced requirement for binding [18,19]. Therefore it is likely that multiple Gαi/o GPCRs may serve as sources for striatal Gβγ. Data from iMSNs present an illustrative model: dopamine activation of D2R→Gαi/o simultaneously inhibits AC5 (via Gαi) and releases Gβγ (from Gαo) to be sequestered by KCTD5. Therefore the net effect at the cyclase level is inhibition. However, in the absence of KCTD5, Gβγ is available to sensitize the cyclase for concurrent stimulatory inputs from Gαolf-coupled receptors such as A2AR. The result in KCTD5 KO is cyclase sensitization. Indeed A2AR stimulation significantly enhances cAMP response in the absence of KCTD5. Accordingly, we report higher levels of baseline cAMP in iMSNs that are rescued by chronic A2AR inhibition. Moreover, D2R signaling resulted in greater cAMP inhibition in the absence of KCTD5. As opposed to dMSNs, iMSNs selectively express two Gαolf-coupled receptors that promote cAMP: (i) constitutively active GPR6 [68,69], (ii) Adenosine 2A Receptor (A2AR) [37]. As iMSNs exhibit higher baseline cAMP and excitability relative to dMSNs [17,33], it is conceivable that loss of KCTD5 would greatly elevate the baseline cAMP through Gβγ sensitization of AC5. Starting at an elevated baseline would enable inhibitory D2R signaling to allow a larger pool of cAMP to be degraded by phosphodiesterases after turning off cyclase enzymatic activity. This mechanism is further supported by evidence that the sensitivity of evoked D2R was unchanged between WT and iMSN KO. In our model, tonic background dopamine would not greatly reduce cAMP, which would facilitate the higher basal cAMP level via signaling from Gαolf-coupled receptors.

In dMSNs, which do not express D2R, attractive candidates for Gβγ sensitization include the M4 muscarinic acetylcholine receptor or the A1 adenosine receptor. The M4R on dMSNs in particular was reported to provide crosstalk to stimulatory D1R signaling [17], markers for cholinergic transmission have one of the highest levels in the striatum [70–72], and acetylcholine has been implicated in motor dysfunction [73]. Alternative considerations in both MSNs could include proteins that contain the Gα-binding-and-activating (GBA) motif such as GIV or DAPLE, which have been reported to enable Gβγ-effector interactions in the absence of GPCRs [74]. However, interplay between GBA proteins and KCTD5 has not yet been investigated. Altogether, KCTD5 likely operates at the downstream nexus from GPCRs connecting crosstalk between G proteins with effectors.

Collectively, our results support a striatal role for KCTD5 in motor coordination likely driven by influence over transduction of neuromodulators including the decoding of dopamine signals. In this light, our *Kctd5* cKO mouse models have utility in future studies toward other aspects of striatal physiology such as reward and aversion. Given the general influences of cAMP sensitization in other areas (discussed above), examination of KCTD5, or redundant isoforms, expressed in other tissues may further expand the portfolio of information on KCTD in vivo. For instance, KCTD5 has recently been implicated in cancer biology [75], which may present an additional arena of exploration for prospective studies.

## Methods

### Animal models

Experimental protocols were approved by Augusta University’s Institutional Animal Care and Use Committee (Animal Use Protocol 2020-1036) and Institutional Biosafety Committee (Biosafety Protocol #2037). Mice were housed under standard conditions on a 12-hour light/dark cycle with unlimited access to both food and water. The following previously described strains were utilized: (i) *cAMP E*ncoded *R*eporter (*CAMPER*) (C57BL/6-Gt (ROSA) 26Sortm1 (CAG-ECFP*/Rapgef3/Venus*)Kama/J) (RRID:IMSR_JAX:032205), (ii) *Drd1aCre* (B6.FVB(Cg)- Tg(Drd1-cre)EY262Gsat/Mmucd) (RRID:MMRRC_030989-UCD), (iii) *Adora2aCre* (B6.FVB(Cg)-Tg(Adora2a-cre) KG139Gsat/Mmucd) (RRID:MMRRC_036158-UCD). *Kctd5* conditional knockout allele was generated by a previously established CRISPR/Cas9 method (Transgenic and Genome Editing Core Facility, Augusta University) [76]. sgRNA targeting the 5′ (5′-TACTTACGGACGGTCACTAC-3′) and 3′ (5′-GACCTGATCTGCCAGTGCAA-3′) of *Kctd5* Exon 3 were used with single-stranded oligonucleotide (ssODN) repair templates that contained loxP sites (5′ ssODN: 5′-AGTATCTGTGGGCTGAGACCCGGTATCCTTCCTCCGTGTGTCACCTGgAtccataacttcgtatagcatacattatacgaagttatGTGACCGTCCGTAAGTATGTCTAGTGAACTGCCTGGCATTTTCAGTCCTATGCT-3′ and 3′ ssODN: 5′-AGATGCTGGAGAGCAGAGGCTCTGATGGGAGAATATCAAGACCTGATCTGCCAGTGataacttcgtatagcatacattatacgaagttatCtcgAGGCCCATGGCCAGCACAGCAGAGAGGAGAGCATCGAGGAGCTGGGAAGTAGGT-3′). Reagents were injected into C57BL/6J pronuclear-stage zygotes and transferred into pseudopregnant recipient female mice to obtain founder pups. Standard PCR was utilized to verify loxP sites with the following primer sets: 5′ loxP verification: FW: 5′-CAGGCACCTTCCAAGCTGCA-3′, RV: 5′-CCTGAGGTTCTTGTGTCAGGCT-3′; 3′ loxP verification: FW: 5′-GCTGGGGAGAGTGAAGATGCT-3′, RV: 5′-CACACATGCTGGGCAGCAGA-3′. The *Kctd5* cKO founder was crossed with C57BL/6J and germline transmission confirmed via Sanger sequencing. Mice were then backcrossed for five generations before performing experiments. DNA samples from ear punches were utilized for genotyping through standard PCR protocols established for every strain in the study. Behavioral testing was performed with the investigator genotype blinded to mice between 2 and 4 months of age. Both male and female mice were used for experiments. SCH 23390 hydrochloride (Cayman Chemical # 15631) stock solution was prepared in in sterile 0.9% NaCl. Istradefylline (Tokyo Chemical Industry #I1100) stock solution was prepared in DMSO for subsequent dilution in sterile 0.9% NaCl. Warmed suspension was injected in mice (intraperitoneal; I.P.) with identical DMSO concentration in vehicle control.

### Accelerating forward rotarod

Mice were placed on a custom-engineered device with 4-inch barrel diameter rod as previously described [77]. The rotarod accelerated from 4 to 60 rpm over 5 min and latency to fall into catch basins was recorded. If a mouse clung to the rod for one complete revolution the trial ended, and that time point was used as the latency. Three trials were performed each day with a 5-min rest period in between. Mice were then tested for five consecutive days. Learning rate was calculated by subtracting the mean latency on day 5 from the mean latency on day 1, which was then divided by the number of days (5), as similarly reported [78].

### Hindlimb clasping

Mice were held suspended by the tail for 30 s while observing hindlimb action patterns and scored as previously reported [79]. The maximum pathological score (4) was assigned if both limbs were clasped (or retracted toward the body) for more than 15 s. A score of 3 was assigned if both hindlimbs were observed to exhibit clasping at any time. A score of 2 was assigned if one of the hindlimbs was clasped for over 15 s. A score of 1 was assigned if one of the hindlimbs was clasped at any time. A score indicating no pathology (0) was assigned in both limbs were extended outward an unclasped during the 30-s period.

### Reverse rotarod

Mice were situated on the rotarod in reverse orientation with a barrier that inhibited turning or climbing but enabled walking backward, as similarly reported [17,20]. Following a 5-min acclimation period, rotarod acceleration commenced at a rate 4–60 rpm over a 5-min period. The latency to fall for each mouse was recorded and we did not observe any mice cling to the rod for a complete revolution. Three trials were performed each day with a 20-min rest period in between trials.

### Open-field assessment

Mice are placed in an open box (10.75″ × 10.75″ × 8″ H (27 × 27 × 20.3 cm) where distance traveled and time spent in center versus perimeter was recorded (Med Associates Inc. ENV-510, Activity Monitor 5 and 7). Mice were allowed to explore for 30 min and data resolution was split into 5-min bins. The arena was cleaned between animals.

### Grip strength

Force was measured using a rodent grip strength meter (Ugo Basile). The mouse was positioned to hold the metal bar while tail-suspended and gently pulled back until releasing the bar. The distance of bar pulled before released from the mouse’s paws is measured in kilograms of force. The average of two independent readings was reported for each mouse.

### Keypoint motion sequencing

As previously reported [80], Keypoint-MoSeq was used to identify the modular syllables that compose sequences of spontaneous behavior. Mice were individually recorded from above for 1 hour with a Google Pixel 7a camera in a rectangular acrylic box (52 × 52 cm base, 73 cm high) with white, opaque walls and flooring. Recordings were cropped to a 1:1 aspect ratio and downsampled to a resolution of 720p. Markerless keypoint tracking was performed using DeepLabCut (version 2.3.9) [81] with the pretrained SuperAnimal-TopViewMouse model [82]. Keypoint-MoSeq models were fit with the resulting keypoint data. Code is publicly available on our Github page (https://github.com/BrianMunteanResearch/KCTD5_MoSeq_Analysis) and archived on Zenodo (https://doi.org/10.5281/zenodo.15019085).

### Primary striatal culture

As similarly reported [33], pups at day postnatal day 0 (P0) from homozygous *Kctd5* cKO breeding pairs were decapitated and the brain rapidly extracted to dissect whole striata in ice-cold HBSS containing 20% Fetalgro (RMBio), NaHCO3 (4.2 mM), and HEPES (1 mM). Striata were washed in HBSS without Fetalgro and digested for 15 min at 37°C in a pH 7.2 buffer containing (in mM): NaCl (137), KCl (5), Na2HPO4 (7), Hepes (25), and 0.3 mg/ml papain (Worthington). Striata were then washed with HBSS (20% Fetalgro), HBSS (no Fetalgro), and growth media (Neurobasal-A containing 2 mM GlutaMAX, B27 supplement, 100 units/ml penicillin, and 100 μg/ml streptomycin). A P1000 pipette was then used to triturate striata in growth media supplemented with DNase I (0.05 U/μl, Invitrogen #18,047,019). Neurons were then plated on poly-D-lysine (PDL; Millipore Sigma #P6407) coated German glass coverslips and maintained at 37°C in a humidified incubator with 5% CO_2_. Half media changes were performed after 2 days from plating and every 3 days thereafter until harvest for western blot. AAV infection was performed as indicated in the text.

### Western blot

Primary cultures were scraped from coverslips in ice-cold lysis buffer containing 1X PBS supplemented with 150 mM NaCl, 1% Triton-X, and protease inhibitor (Thermo Fisher Scientific #A32955). Dorsal striatal tissue punches (2 mm) were homogenized in lysis buffer supplemented with Phosphatase Inhibitor Cocktail (Cell Signaling Technology 5870). After 15-s sonication at 30% power (FisherBrand #FB50110), the sample was frozen at −80°C for 30 min, and total lysate was collected after centrifugation at 12,5000 rpm for 10. Sample concentration was determined using the Pierce 660 nm reagent (Thermo Fisher Scientific #22660) and samples were diluted to identical concentrations in an SDS-based buffer. Proteins were denatured by heating at 37°C for 15 min. Twenty micrograms of total protein was then resolved via SDS-PAGE, transferred to PVDF membranes, and blocked with 5% dry non-fat milk (LabScientific #M0841) in PBS containing 0.1% Tween-20 (PBST). Primary (anti-KCTD2/5: ProteinTech 15553–1-AP, anti-β-Actin (8H10D10): Cell Signaling Technology 3700S, anti-DARPP-32 (19A3): Cell Signaling Technology 2306S, anti-Phospho-DARPP-32 (T34): R&D Systems AF2847, anti-GluA1 (D4N9V): Cell Signaling Technology 13185S, anti-GluA1 (Ser845) (D10G5): Cell Signaling Technology 8084S) and secondary (anti-mouse-HRP IgG light chain: Jackson ImmunoResearch 115-035-174, anti-rabbit-HRP IgG light chain: Jackson ImmunoResearch 211-032-171) antibodies were sequentially applied in 1% milk-PBST solution. Protein bands were imaged by membrane exposure to enhanced chemiluminescence reagent followed by digital capture with the KwikQuant Imager (Kindle Biosciences #D1001). Band intensity was quantified using standard ImageJ tools.

### cAMP ELISA

Total cAMP from dorsal striatal brain punches (2 mm) were measured using an ELISA kit (ENZO Life Sciences #ADI-901–066), as previously reported [83–85]. Tissue was weighed then homogenized and diluted in 0.1 M HCl. Absorbance was recorded on SpectraMax microplate reader (Molecular Devices).

### Acute brain slice preparation

As previously reported [33,34,49] adult mice (2–3 months) were anesthetized with isoflurane, decapitated, and whole brain rapidly mounted on a vibratome (Precisionary VF-310-0Z) in ice-cold oxygenated buffer consisting of (in mM): KCl (2.5), NMDG (93), glucose (25), HEPES (20), sodium ascorbate (5), sodium pyruvate (3), thiourea (2), NaH2PO4 (1.2), CaCl2 (0.5), MgCl2 (10), NaHCO3 (30). Coronal brain slices containing striatum were sectioned at 300 micrometer thickness and incubated for 1 hour at 34°C in an oxygenated recovery buffer consisting of (in mM): NaCl (126), KCl (2.5), CaCl2 (2), MgCl2 (2), NaHCO3 (18), NaH2PO4 (1.2), and glucose (10).

### 2-photon FRET imaging

Imaging experiments were performed on a Zeiss 780 multiphoton confocal microscope through a 20X W Plan-Apochromat objective. A Ti:Sapphire laser (Coherent Chameleon Vision S) tuned to 850 nm was utilized for FRET donor excitation. XYZ image stacks were captured at 15-s intervals from emission of FRET donor (mTurquoise: 455–500 nm) and acceptor (Venus: 526–571 nm) simultaneously through dual photomultiplier tubes. The dorsal striatum of the brain slices was imaged in a recording chamber (Warner Instruments) that enabled constant perfusion (~2 ml/min) of an oxygenated buffer that consisted of (in mM): NaCl (125), KCl (2.5), CaCl2 (2), MgCl2 (2), NaH2PO4 (1.25), NaHCO3 (25), and glucose (25). A DS3 isolated current stimulator (Digitimer) was controlled by Pulser (Prizmatix) software to enable electrical stimulation (20 ms pulse; 650 μA) by inserting a tungsten microelectrode (World Precision Instruments) adjacent to the field of view. Evoked dopamine responses were aided by supplementing the recording buffer with the following antagonists, as previously reported [86]: Picrotoxin (100 μM), CGP 55,845 (300 nM), DNQX (10 μM), Scopolamine (250 nM), DPCPX (1 μM). FRET values were calculated from raw intensity changes of the neuron cell body utilizing ImageJ tools, as previously reported [33,34,49]. As indicated in the text for some experiments, sign of cAMP response to dopamine was utilized to classify MSN subtype (dMSN increased cAMP; iMSN decreased cAMP).

### Molecular cloning

pAAV-hSyn-Cre-P2A-dTomato (plasmid #107738) and pAAV-CAG-tdTomato (plasmid #59462) were obtained from Addgene. Nanobody 5 (Nb5) and Nanobody 17 (Nb17) were previously reported ^36^ and codon-optimized open reading frame were synthesized (Twist Biosciences) with PspXI and HindIII restriction enzyme sites. Nb inserts were then cloned into the PspXI/HindIII sites on pAAV-hSyn-Cre-P2A-dTomato in frame to generate: pAAV-hSyn-Cre-P2A-Nb5 and pAAV-hSyn-Cre-P2A-Nb17. Constructs were verified by whole plasmid sequencing (Plasmidsaurus).

### AAV packaging and purification

AAV particles were obtained from Human embryonic kidney cells (Lenti-X HEK293T; Takara Bio #632180). Growth media for HEK293 cells consisted of DMEM (Gibco #11995) supplemented with 7.5% Fetalgro EX (RMBio; FGX-BBT), minimum Eagle’s medium non-essential amino acids, 100 units/ml of penicillin, and 100 μg/ml of streptomycin. Cells were maintained in a humidified incubator at 37°C with 5% CO2. AAV packaging was performed by triple transfection of pAAV2/5 (Addgene plasmid #104964), pAdDeltaF6 (Addgene plasmid #112867), and AAV of interest (above). Cells were plated on PDL (Gibco #A38904) coated dishes for transfection via polyethylenimine (Polysciences #23,966−100)/OptiMEM (Gibco #11058021) and 24 hours after transfection the media was replaced with OptiPro SFM (Gibco #12,30019) as similarly reported [87]. Seventy-two hours post-transfection, AAV particles were then obtained by a previously reported extraction protocol, and viral titers were assessed by PCR [88]. AAVs were kept in glass vials at +4°C in at ~10^13^ GC/ml and used within a month for experiments.

### Stereotaxic injections

As previously reported [89], neonatal pups (P0-P1) were thermally anesthetized by ice for five minutes followed by placement on a rodent stereotaxic frame (Stoelting) in custom 3D-printed device that enabled continued ice anesthesia [90]. The dorsal striatum was then targeted for delivery of AAV particles. A Hamilton syringe coupled to a 30-gauge needle was carefully lowered into the brain (AP: +2.4 mm anterior to lambda, ML ± 1.0 mm, DV −1.7 mm) via manipulator control [49]. A total volume of 200 nl of AAV particles was bilaterally infused over a 4-min period. The needle tip was left in place for one additional minute before slowly removing. Pups then recovered in a 33°C incubator for 30 min before returning to their home cage.

### Statistical information

Statistical analysis was performed using GraphPad Prism 10 with exception of Keypoint MoSeq results. Data are presented as the mean ± standard error of the mean unless otherwise indicated in the text. The number of biological replicates, statistical tests performed, and p values are provided for each experiment in the appropriate figure legend.

## Supporting information

S1 Fig Striatal Kctd5 knockout mouse characterization. **(A**) Scheme of Mus musculus Kctd5 gene. **(B)**. Sanger sequencing of founder *Kctd5* conditional knockout allele verifying presence of loxP insertion that flanks exon 3. **(C)** Identification of *Kctd5 cKO* allele through standard PCR-agarose gel genotyping. **(D)** Western blot detection of KCTD5 and Actin from *Kctd5*^*cKO/cKO*^ primary striatal neuron cultures after 14 days of infection with either Control (AAV-dTomato) or Cre (AAV-Cre-P2A-dTomato) AAV particles. **(E)** Quantification of KCTD5 protein level from primary culture western blot. *n* = 3 primary cultures, unpaired *t* test, *p* = 0.0253. **(F)** Body weight measurement of *Kctd5* cKO mice at 3 months of age. dMSN male: KO (*n* = 8) and WT (*n* = 8), dMSN female: KO (*n* = 8) and WT (*n* = 8), iMSN male: KO (*n* = 8) and WT (*n* = 8), iMSN female: KO (*n* = 7) and WT (*n* = 9). All data presented as mean ± SEM. The data underlying this figure can be found in S1 Raw Images. The numerical data presented in this figure can be found in S2 Data.
(TIF)

S2 Fig Striatal Kctd5 knockout mouse grip strength and open-field analysis. **(A**) Grip strength quantification in Kctd5 cKO. dMSN male, KO (*n* = 7) and WT (*n* = 8), nonparametric *t* test, Mann–Whitney *U* = 25, *p* = 0.7559. dMSN female, KO (*n* = 8) and WT (*n* = 9), nonparametric *t* test, Mann–Whitney *U* = 24, *p* = 0.2653. iMSN male, KO (*n* = 5) and WT (*n* = 5), nonparametric *t* test, Mann–Whitney *U* = 10, *p* = 0.6508. iMSN female, KO (*n* = 7) and WT (*n* = 8), nonparametric *t* test, Mann–Whitney *U* = 18, *p* = 0.2671. (**B**) dMSN KO and WT comparison of total distance traveled in the open-field arena. Male, KO (*n* = 7) and WT (*n* = 8), nonparametric *t* test, Mann–Whitney *U* = 19, *p* = 0.3357. Female, KO (*n* = 8) and WT (*n* = 10), nonparametric *t* test, Mann–Whitney *U* = 26, *p* = 0.2370. **(C)** iMSN KO and WT comparison of total distance traveled in the open-field arena. Male, KO (*n* = 5) and WT (*n* = 7), nonparametric *t* test, Mann–Whitney *U* = 12, *p* = 0.4318. Female, KO (*n* = 7) and WT (*n* = 10), nonparametric *t* test, Mann–Whitney *U* = 28, *p* = 0.5362. **(D)** Quantification of zone entries in the open-field arena. dMSN male, KO (*n* = 6) and WT (*n* = 8), nonparametric *t* test, Mann–Whitney *U* = 21, *p* = 0.7293. dMSN female, KO (*n* = 8) and WT (*n* = 12), nonparametric *t* test, Mann–Whitney *U* = 32, *p* = 0.2380. iMSN male, KO (*n* = 5) and WT (*n* = 7), nonparametric *t* test, Mann–Whitney *U* = 14, *p* = 0.6389. iMSN female, KO (*n* = 7) and WT (*n* = 11), nonparametric *t* test, Mann–Whitney *U* = 32, *p* = 0.5962. **(E)** dMSN KO and WT comparison of time spent in the center of the open-field arena. Male, KO (*n* = 7) and WT (*n* = 8), nonparametric *t* test, Mann–Whitney *U* = 28, *p* > 0.9999. Female, KO (*n* = 8) and WT (*n* = 12), nonparametric *t* test, Mann–Whitney *U* = 40, *p* = 0.5714. **(F)** dMSN KO and WT comparison of time spent in the corner of the open-field arena. Male, KO (*n* = 7) and WT (*n* = 8), nonparametric *t t*est, Mann–Whitney *U* = 28, *p* > 0.9999. Female, KO (*n* = 8) and WT (*n* = 12), nonparametric *t* tes*t*, Mann–Whitney *U* = 40, *p* = 0.5714. **(G)** iMSN KO and WT comparison of time spent in the center of the open-field arena. Male, KO (*n* = 5) and WT (*n* = 7), nonparametric *t* test, Mann–Whitney *U* = 8, *p* = 0.1490. Female, KO (*n* = 7) and WT (*n* = 11), nonparametric *t* test, Mann–Whitney *U* = 36, *p* = 0.8601. **(H)** iMSN KO and WT comparison of time spent in the corner of the open-field arena. Male, KO (*n* = 5) and WT (*n* = 7), nonparametric *t* tes*t*, Mann–Whitney *U* = 8, *p* = 0.1490. Female, KO (*n* = 11) and WT (*n* = 7), nonparametric *t* tes*t*, Mann–Whitney *U* = 36, *p* = 0.8601. All data presented as mean ± SEM. The numerical data presented in this figure can be found in S2 Data.
(TIF)

S3 Fig Keypoint motion sequencing behavioral syllables in striatal Kctd5 knockout. **(A)** Identified syllables in dMSN (KO: *n* = 6, WT: *n* = 7) and iMSN (KO: *n* = 6 and WT: *n* = 6) cohorts. (**B**) Similarity between individual behavioral syllables. **(C)** Frequency of syllable usage in dMSN KO and dMSN WT. Data presented as mean ± SEM. **(D)** Frequency of syllable usage in iMSN KO and iMSN WT. Data presented as mean ± SEM. **(E)** Heat map of syllable transition in in dMSN KO and dMSN WT. **(F)** Heat map of syllable transition in iMSN KO and iMSN WT. The numerical data presented in this figure can be found in S2 Data. The code for S3A–F Fig is publicly available in a GitHub repository (https://github.com/BrianMunteanResearch/KCTD5_MoSeq_Analysis) and archived on Zenodo (https://doi.org/10.5281/zenodo.15019085).
(TIF)

S4 Fig Characterizing D1R signaling to cAMP in dMSN KCTD5 knockout. **(A**) Average response traces of evoked dopamine (20 Hz, 20 pulses) to a paired-pulse protocol with 30 min between recordings in the presence (24 neurons/6 animals) or absence (28 neurons/6 animals) of SCH23390 (10 μM) in *Drd1a*^*Cre*^:CAMPER^+/+^ slices. (**B**) Maximum cAMP response to 20 Hz paired-pulse stimulation in dMSN. Buffer: Nonparametric *t* test, Mann–Whitney *U* = 312.5, *p* = 0.1956. SCH23390: Nonparametric *t* test, Mann–Whitney *U* = 0, *p* < 0.0001. **(C)** Average response traces to vary stimulation frequencies (20 pulses) in dMSN WT (5 animals, ≥24 neurons/frequency) and dMSN KO (5 animals, ≥27 neurons/frequency). All data presented as mean ± SEM. The numerical data presented in this figure can be found in S2 Data.
(TIF)

S5 Fig Characterizing cAMP signaling in iMSN KCTD5 knockout. **(B**) Average response traces of evoked dopamine (20 Hz, 20 pulses) to a paired-pulse protocol with 30 min between recordings in the presence (22 neurons/5 animals) or absence (19 neurons/4 animals) of Sulpiride (1 μM) in *Adora2a*^*Cre*^*:CAMPER*^+/-^ slices. (**B**) Maximum cAMP response to 20 Hz paired-pulse stimulation in iMSN. Buffer: Nonparametric *t* test, Mann–Whitney *U* = 180, *p* > 0.9999. Sulpiride: Nonparametric *t* test, Mann–Whitney *U* = 0, *p* < 0.0001. (**C)** Average response traces to vary stimulation frequencies (20 pulses) in iMSN WT (6 animals, ≥29 neurons/frequency) and iMSN KO (6 animals, ≥26 neurons/frequency). All data presented as mean ± SEM. The numerical data presented in this figure can be found in S2 Data.
(TIF)

S1 Video Hindlimb clasping example in WT. (MP4)

S2 Video Hindlimb clasping example in KO. (MP4)

S1 Raw Images Raw images of immunoblotting experiments. (PDF)

S1 Data Supporting data for main figures. (XLSX)

S2 Data Supporting data for supplemental figures. (XLSX)

## Abbreviations:

A2AR: adenosine 2A receptor
AC5: adenylyl cyclase type 5
cAMP: cyclic adenosine monophosphate
D1R: Dopamine D1 Receptor
D2R: Dopamine D2 Receptor
GPCR: G protein-coupled receptor
KCTD5: potassium channel tetramerization domain 5
MSN: medium spiny neuron
Nb: Nanobody
