# Supplementary figures and images for: The G protein modifier KCTD5 tunes the decoding of neuromodulatory signals necessary for motor function in striatal neurons
Source: PLoS Biol. 2025 Apr 15;23(4):e3003117. doi: 10.1371/journal.pbio.3003117 (PMC12021292; doi:10.1371/journal.pbio.3003117)

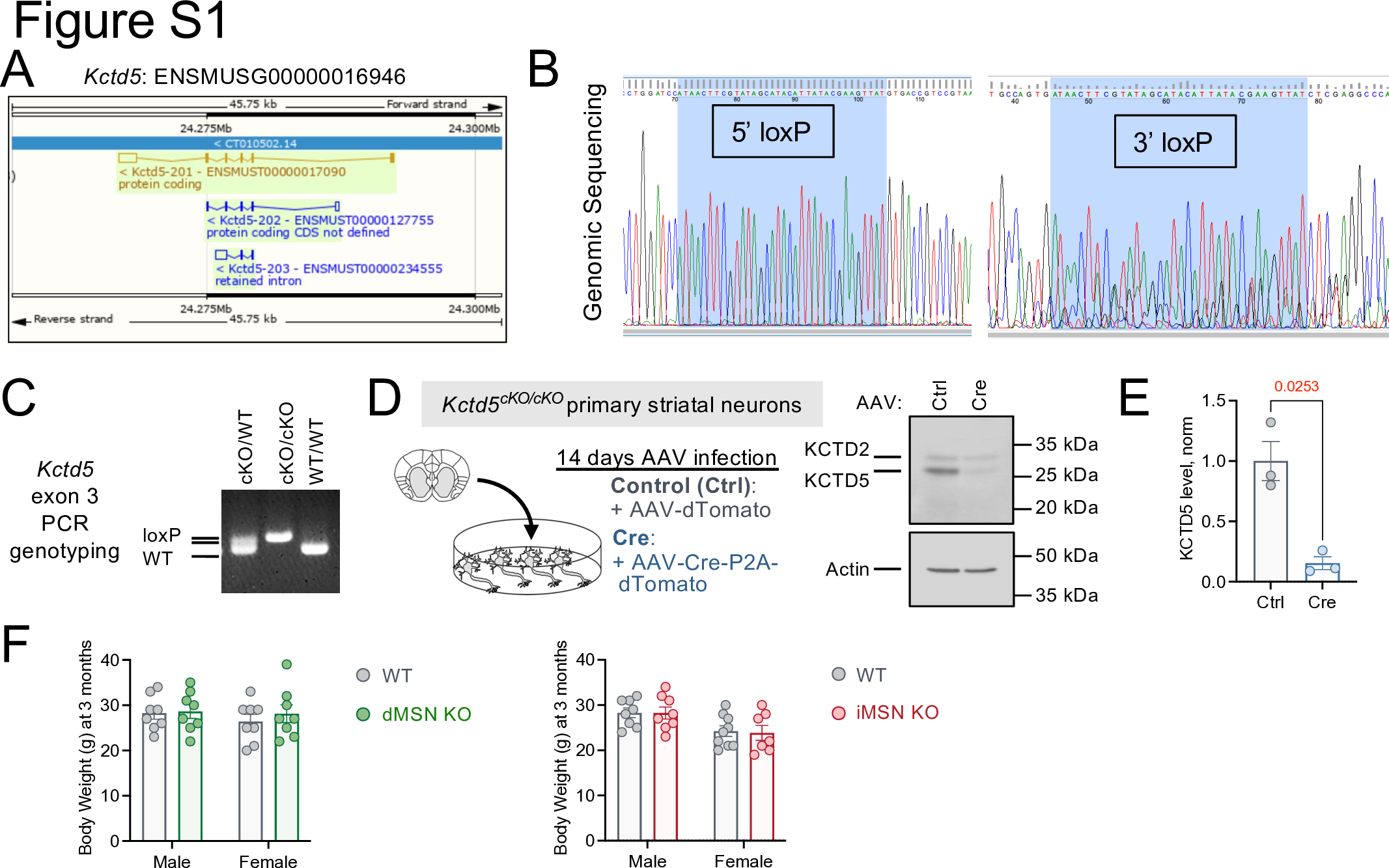

Supplement: S1 Fig — (A) Scheme of Mus musculus Kctd5 gene. (B). Sanger sequencing of founder Kctd5 conditional knockout allele verifying presence of loxP insertion that flanks exon 3. (C) Identification of Kctd5 cKO allele through standard PCR-agarose gel genotyping. (D) Western blot detection of KCTD5 and Actin from Kctd5cKO/cKO primary striatal neuron cultures after 14 days of infection with either Control (AAV-dTomato) or Cre (AAV-Cre-P2A-dTomato) AAV particles. (E) Quantification of KCTD5 protein level from primary culture western blot. n = 3 primary cultures, unpaired t test, p = 0.0253. (F) Body weight measurement of Kctd5 cKO mice at 3 months of age. dMSN male: KO (n = 8) and WT (n = 8), dMSN female: KO (n = 8) and WT (n = 8), iMSN male: KO (n = 8) and WT (n = 8), iMSN female: KO (n = 7) and WT (n = 9). All data presented as mean ± SEM. The data underlying this figure can be found in S1 Raw Images. The numerical data presented in this figure can be found in S2 Data. (TIF) [file pbio.3003117.s001.tif]

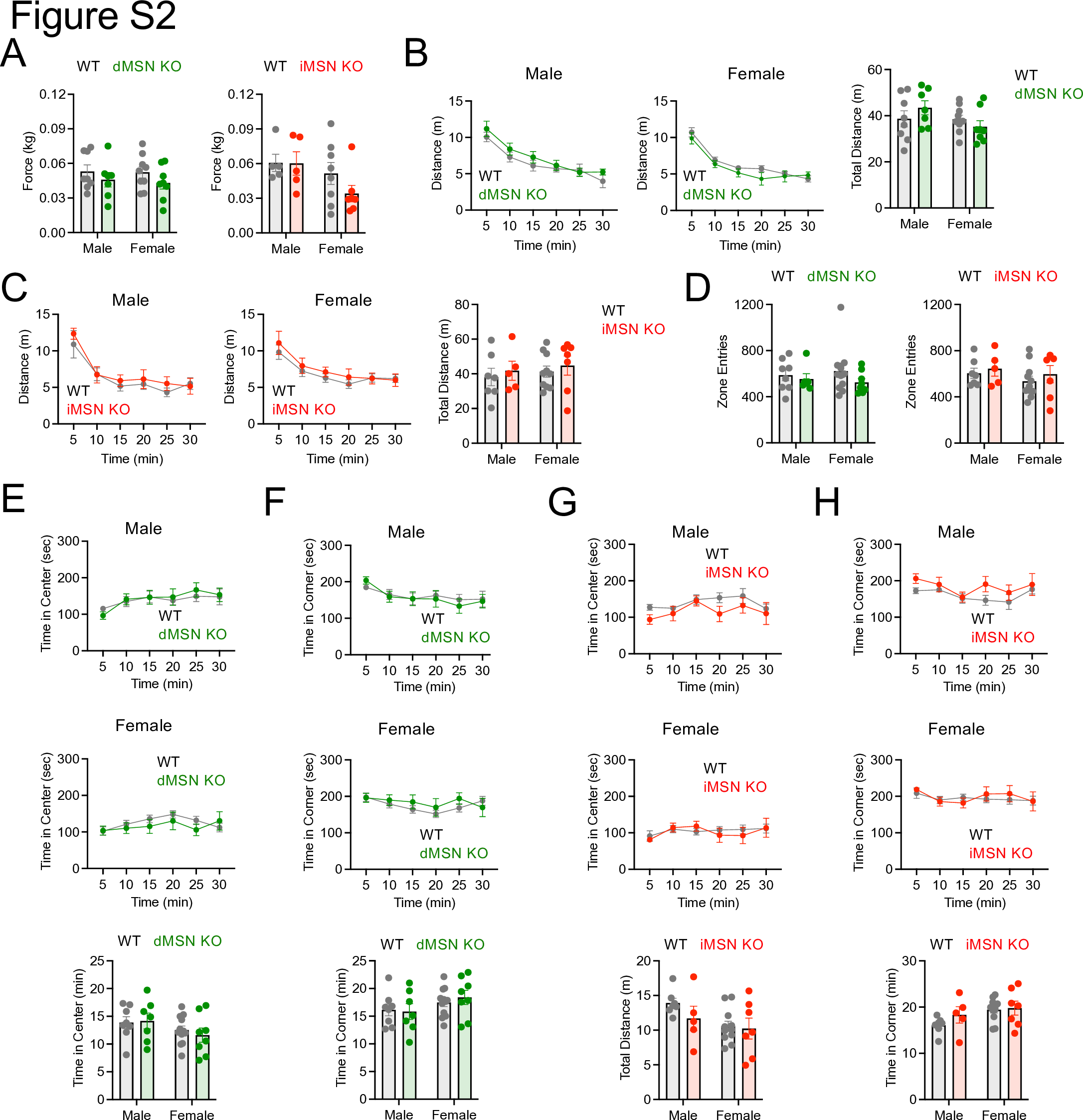

Supplement: S2 Fig — (A) Grip strength quantification in Kctd5 cKO. dMSN male, KO (n = 7) and WT (n = 8), nonparametric t test, Mann–Whitney U = 25, p = 0.7559. dMSN female, KO (n = 8) and WT (n = 9), nonparametric t test, Mann–Whitney U = 24, p = 0.2653. iMSN male, KO (n = 5) and WT (n = 5), nonparametric t test, Mann–Whitney U = 10, p = 0.6508. iMSN female, KO (n = 7) and WT (n = 8), nonparametric t test, Mann–Whitney U = 18, p = 0.2671. (B) dMSN KO and WT comparison of total distance traveled in the open-field arena. Male, KO (n = 7) and WT (n = 8), nonparametric t test, Mann–Whitney U = 19, p = 0.3357. Female, KO (n = 8) and WT (n = 10), nonparametric t test, Mann–Whitney U = 26, p = 0.2370. (C) iMSN KO and WT comparison of total distance traveled in the open-field arena. Male, KO (n = 5) and WT (n = 7), nonparametric t test, Mann–Whitney U = 12, p = 0.4318. Female, KO (n = 7) and WT (n = 10), nonparametric t test, Mann–Whitney U = 28, p = 0.5362. (D) Quantification of zone entries in the open-field arena. dMSN male, KO (n = 6) and WT (n = 8), nonparametric t test, Mann–Whitney U = 21, p = 0.7293. dMSN female, KO (n = 8) and WT (n = 12), nonparametric t test, Mann–Whitney U = 32, p = 0.2380. iMSN male, KO (n = 5) and WT (n = 7), nonparametric t test, Mann–Whitney U = 14, p = 0.6389. iMSN female, KO (n = 7) and WT (n = 11), nonparametric t test, Mann–Whitney U = 32, p = 0.5962. (E) dMSN KO and WT comparison of time spent in the center of the open-field arena. Male, KO (n = 7) and WT (n = 8), nonparametric t test, Mann–Whitney U = 28, p > 0.9999. Female, KO (n = 8) and WT (n = 12), nonparametric t test, Mann–Whitney U = 40, p = 0.5714. (F) dMSN KO and WT comparison of time spent in the corner of the open-field arena. Male, KO (n = 7) and WT (n = 8), nonparametric t test, Mann–Whitney U = 28, p > 0.9999. Female, KO (n = 8) and WT (n = 12), nonparametric t test, Mann–Whitney U = 40, p = 0.5714. (G) iMSN KO and WT comparison of time spent in the center of the open-field arena. Male, KO ( [file pbio.3003117.s002.tif]

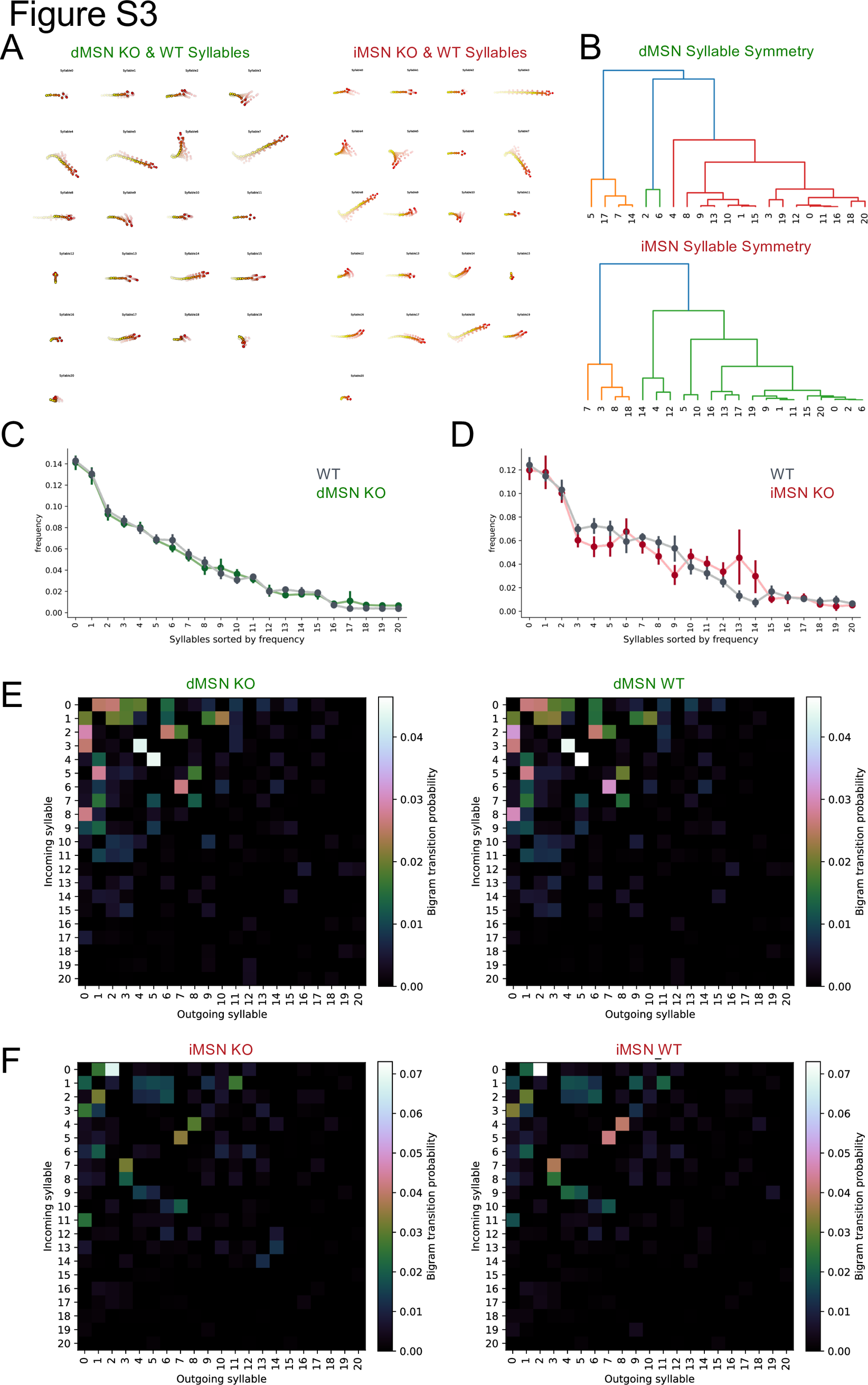

Supplement: S3 Fig — (A) Identified syllables in dMSN (KO: n = 6, WT: n = 7) and iMSN (KO: n = 6 and WT: n = 6) cohorts. (B) Similarity between individual behavioral syllables. (C) Frequency of syllable usage in dMSN KO and dMSN WT. Data presented as mean ± SEM. (D) Frequency of syllable usage in iMSN KO and iMSN WT. Data presented as mean ± SEM. (E) Heat map of syllable transition in in dMSN KO and dMSN WT. (F) Heat map of syllable transition in iMSN KO and iMSN WT. The numerical data presented in this figure can be found in S2 Data. The code for S3A–F Fig is publicly available in a GitHub repository (https://github.com/BrianMunteanResearch/KCTD5_MoSeq_Analysis) and archived on Zenodo (https://doi.org/10.5281/zenodo.15019085). (TIF) [file pbio.3003117.s003.tif]

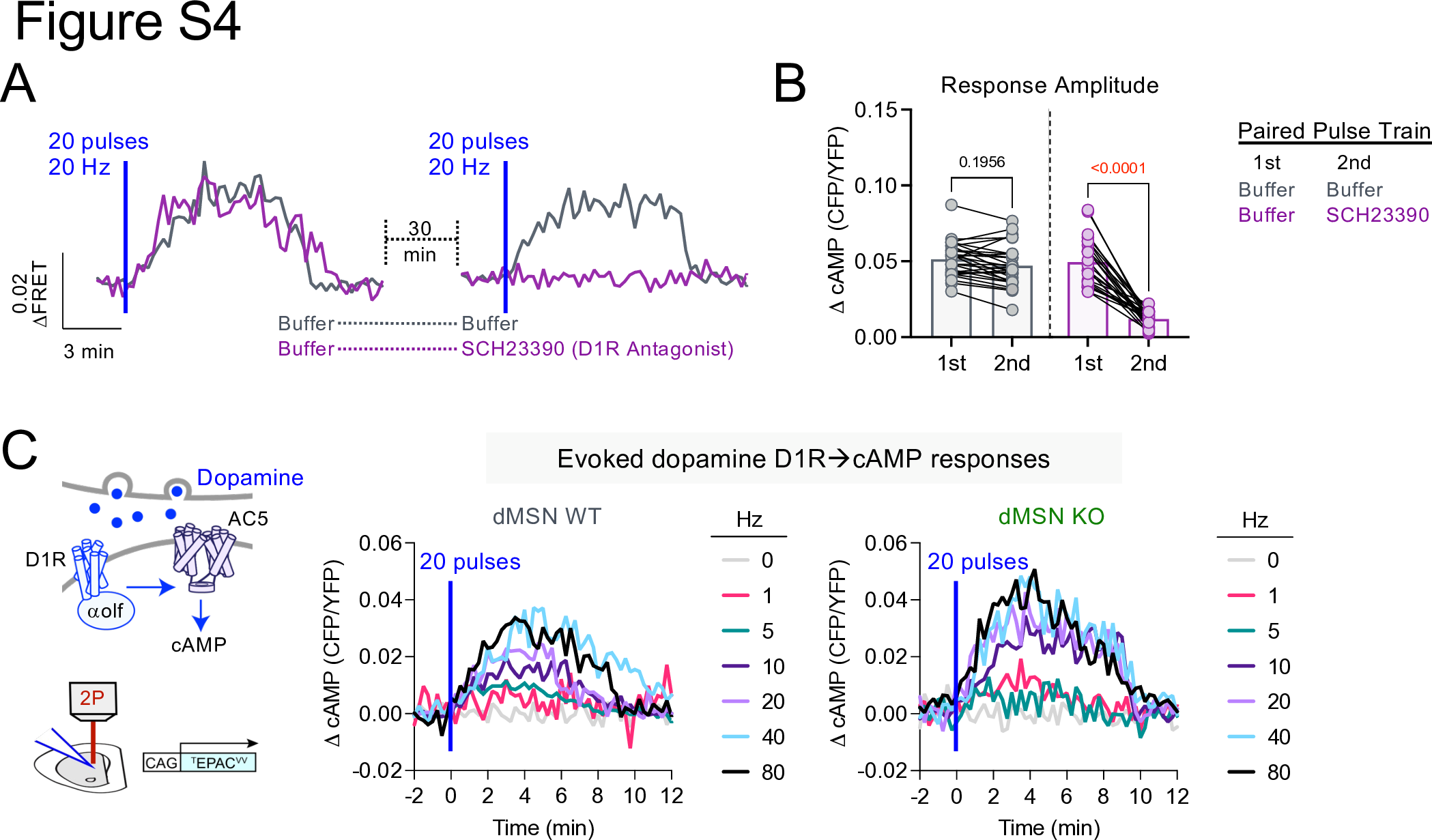

Supplement: S4 Fig — (A) Average response traces of evoked dopamine (20 Hz, 20 pulses) to a paired-pulse protocol with 30 min between recordings in the presence (24 neurons/6 animals) or absence (28 neurons/6 animals) of SCH23390 (10 μM) in Drd1aCre:CAMPER+/+ slices. (B) Maximum cAMP response to 20 Hz paired-pulse stimulation in dMSN. Buffer: Nonparametric t test, Mann–Whitney U = 312.5, p = 0.1956. SCH23390: Nonparametric t test, Mann–Whitney U = 0, p < 0.0001. (C) Average response traces to vary stimulation frequencies (20 pulses) in dMSN WT (5 animals, ≥24 neurons/frequency) and dMSN KO (5 animals, ≥27 neurons/frequency). All data presented as mean ± SEM. The numerical data presented in this figure can be found in S2 Data. (TIF) [file pbio.3003117.s004.tif]

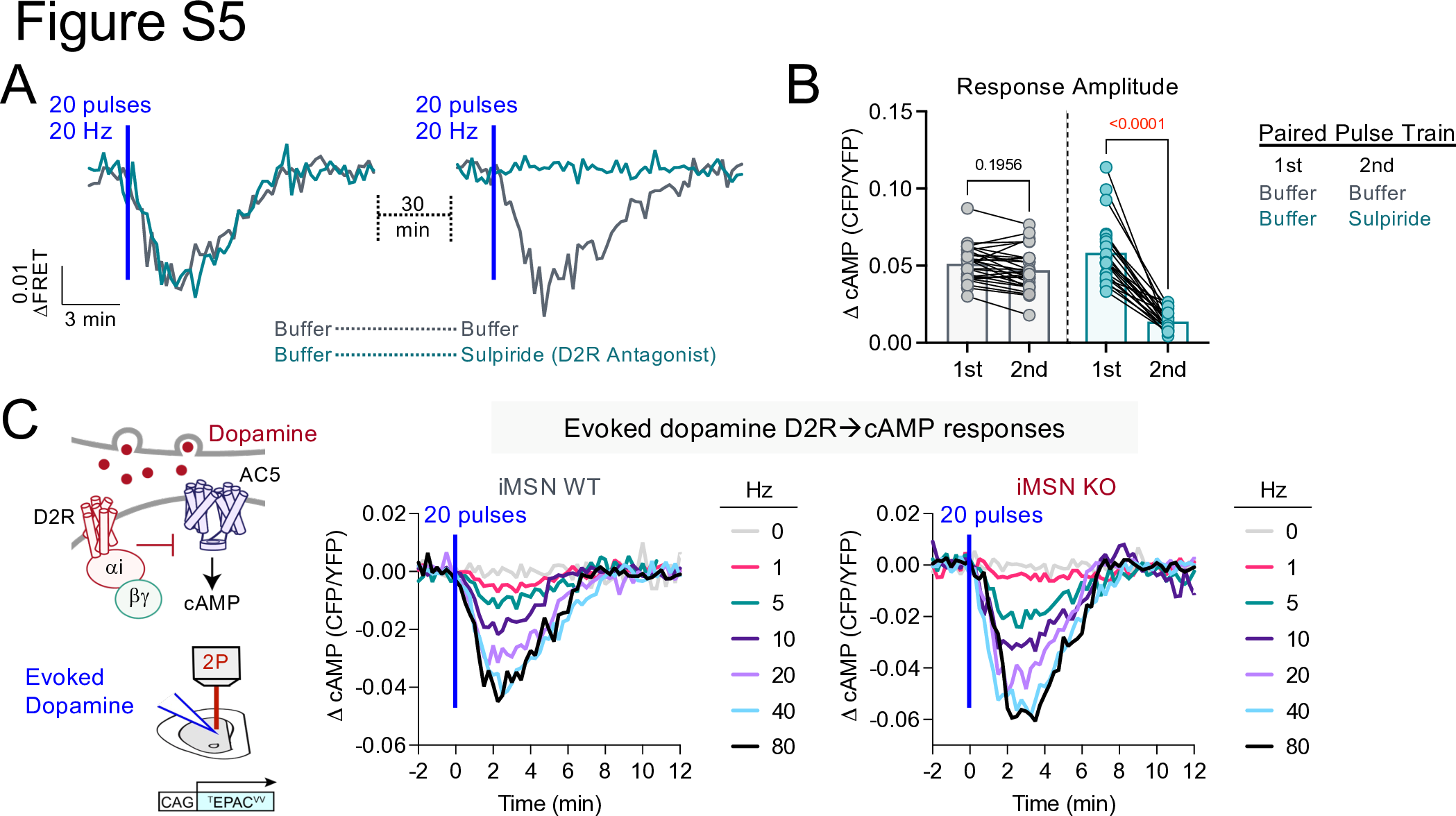

Supplement: S5 Fig — (B) Average response traces of evoked dopamine (20 Hz, 20 pulses) to a paired-pulse protocol with 30 min between recordings in the presence (22 neurons/5 animals) or absence (19 neurons/4 animals) of Sulpiride (1 μM) in Adora2aCre:CAMPER+/- slices. (B) Maximum cAMP response to 20 Hz paired-pulse stimulation in iMSN. Buffer: Nonparametric t test, Mann–Whitney U = 180, p > 0.9999. Sulpiride: Nonparametric t test, Mann–Whitney U = 0, p < 0.0001. (C) Average response traces to vary stimulation frequencies (20 pulses) in iMSN WT (6 animals, ≥29 neurons/frequency) and iMSN KO (6 animals, ≥26 neurons/frequency). All data presented as mean ± SEM. The numerical data presented in this figure can be found in S2 Data. (TIF) [file pbio.3003117.s005.tif]
