# Supplementary material for: The G protein modifier KCTD5 tunes the decoding of neuromodulatory signals necessary for motor function in striatal neurons
Source: PLoS Biol. 2025 Apr 15;23(4):e3003117. doi: 10.1371/journal.pbio.3003117 (PMC12021292; doi:10.1371/journal.pbio.3003117)

Figure S1D

Kctd5 cKO primary neurons
Anti-Kctd5

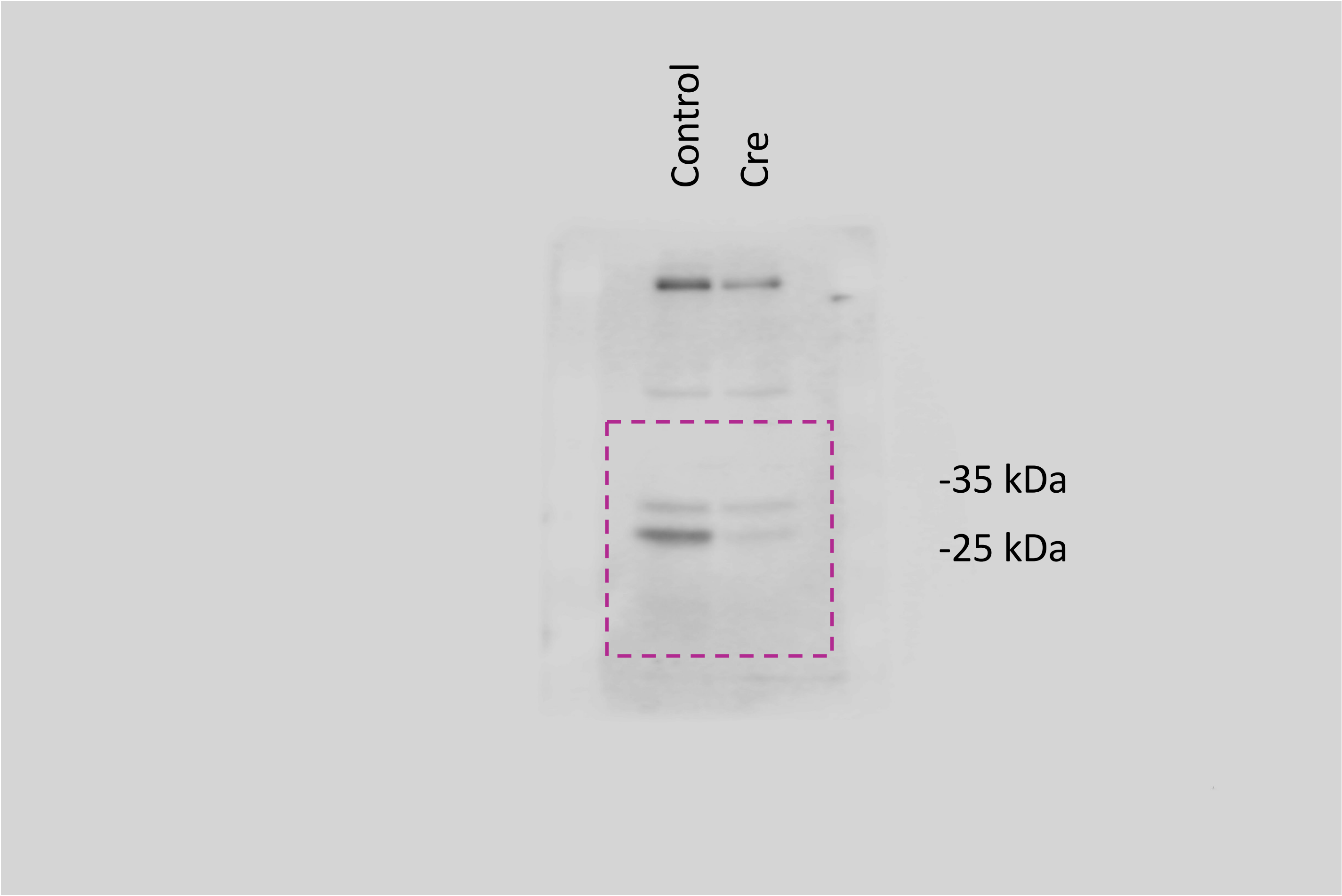

Figure S1D

Kctd5 cKO primary neurons
Anti-Actin

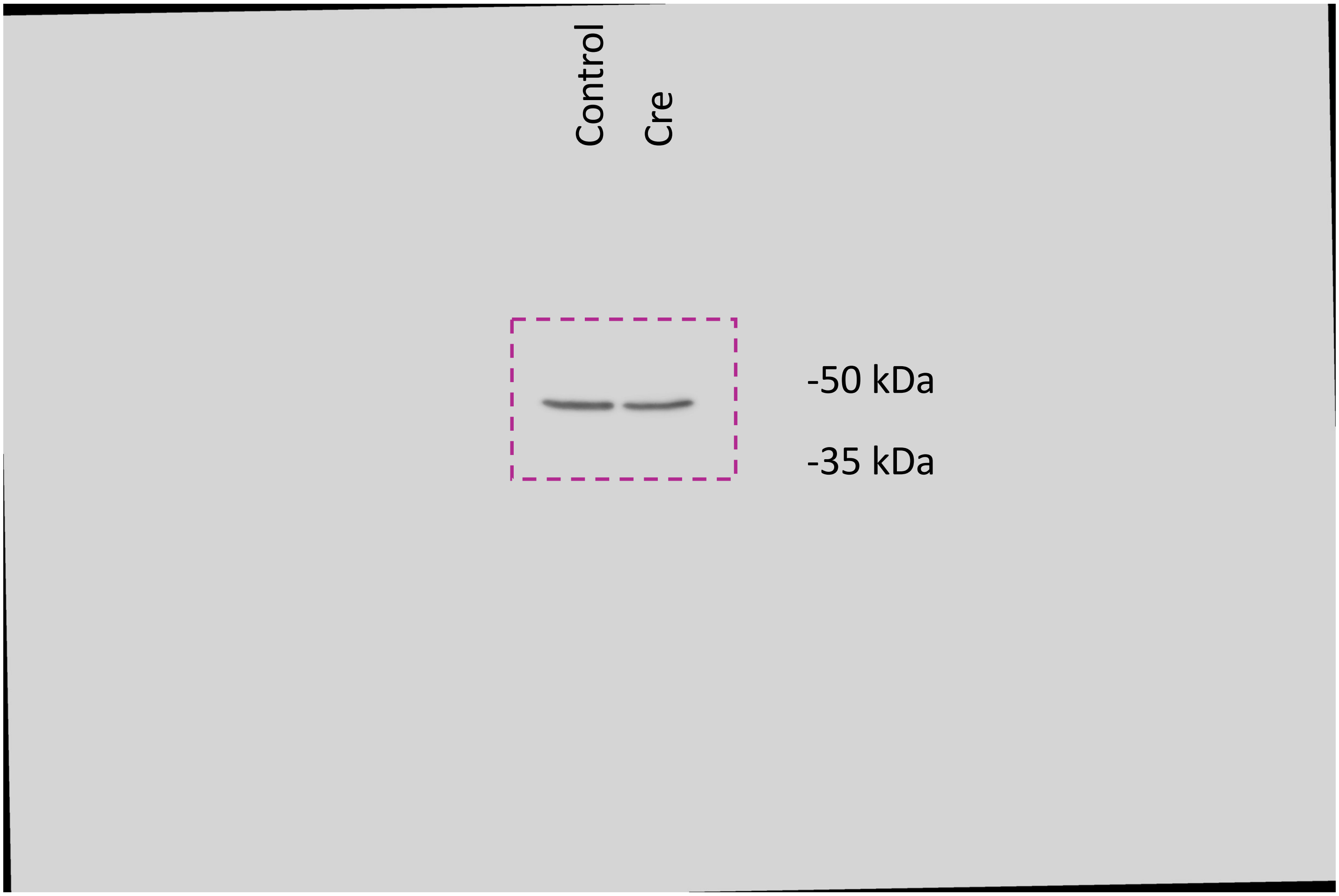

Figure 2B

dMSN KO and dMSN WT
Anti-P-Thr34-DARPP32

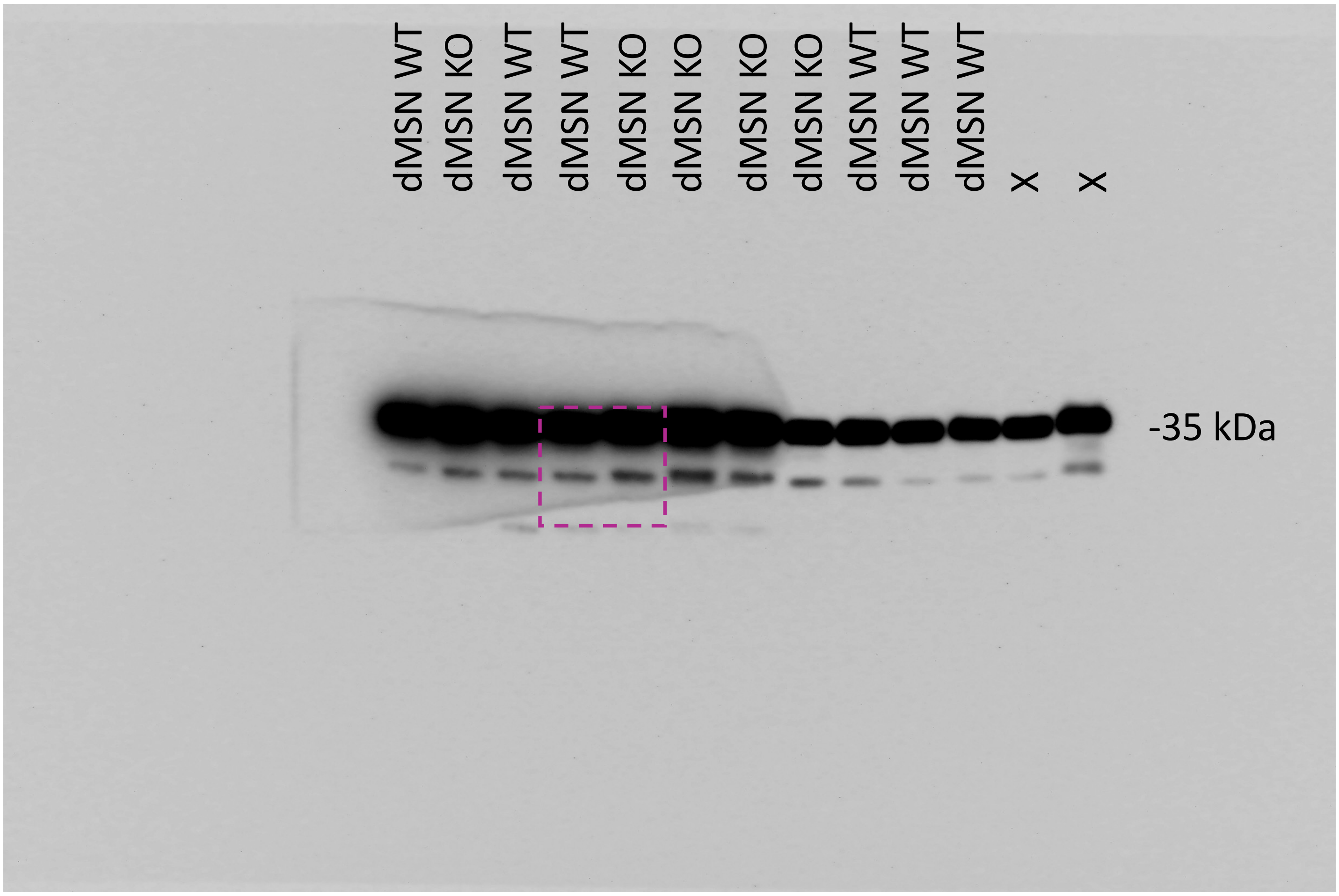

Figure 2B

dMSN KO and dMSN WT
Anti-DARPP32

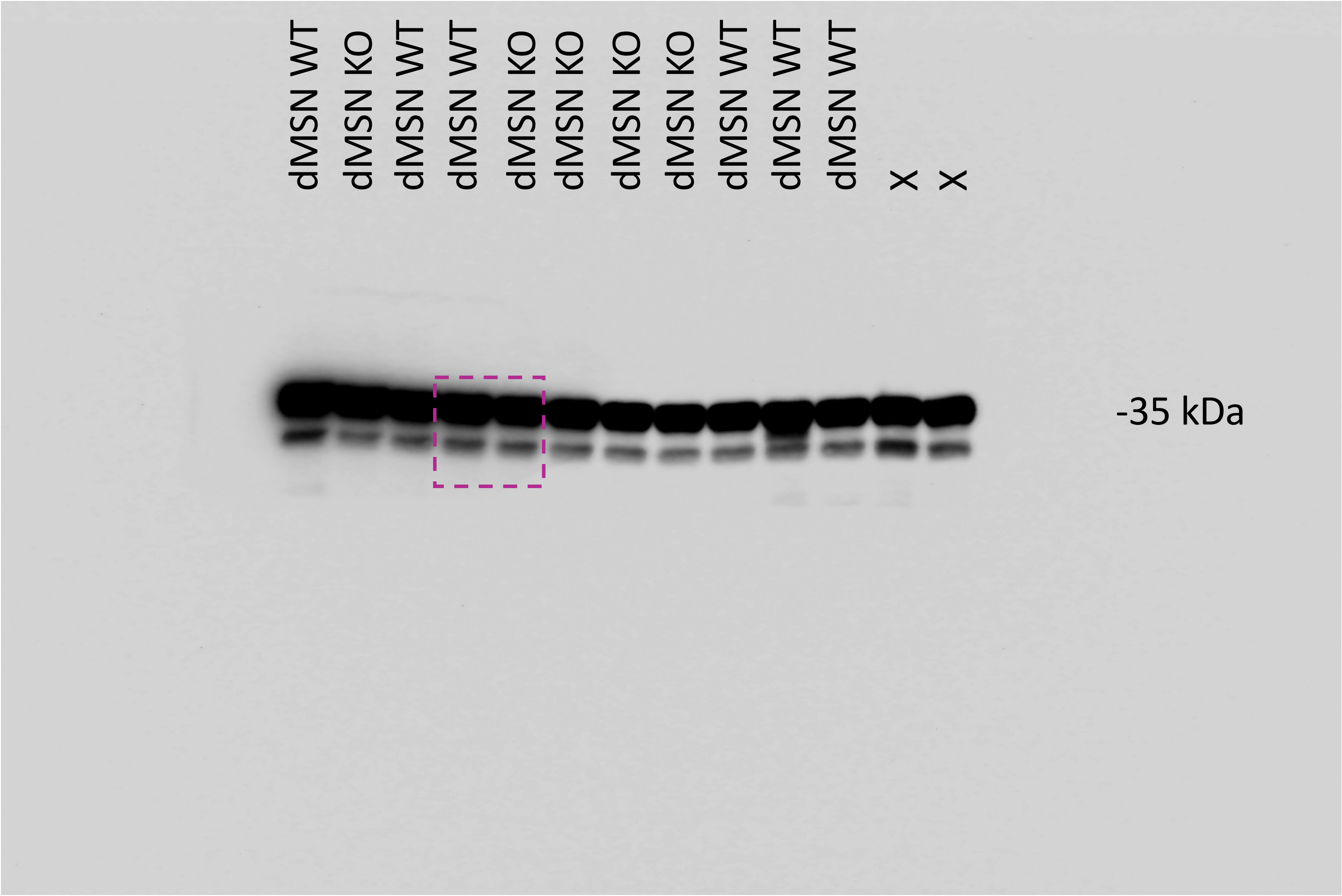

Figure 2B

dMSN KO and dMSN WT
Anti-P-Ser845-GluA1

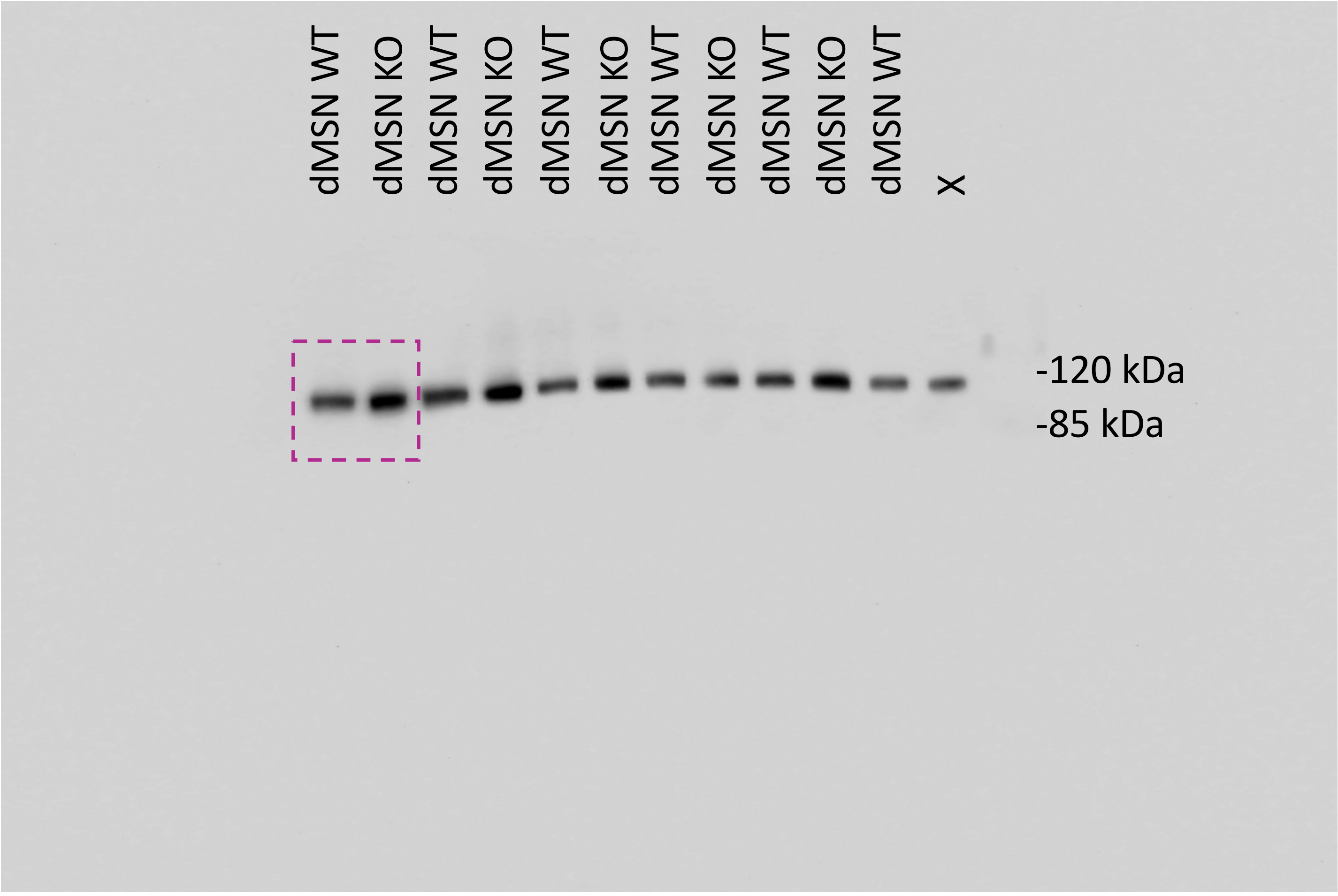

Figure 2B

dMSN KO and iMSN WT
Anti-GluA1

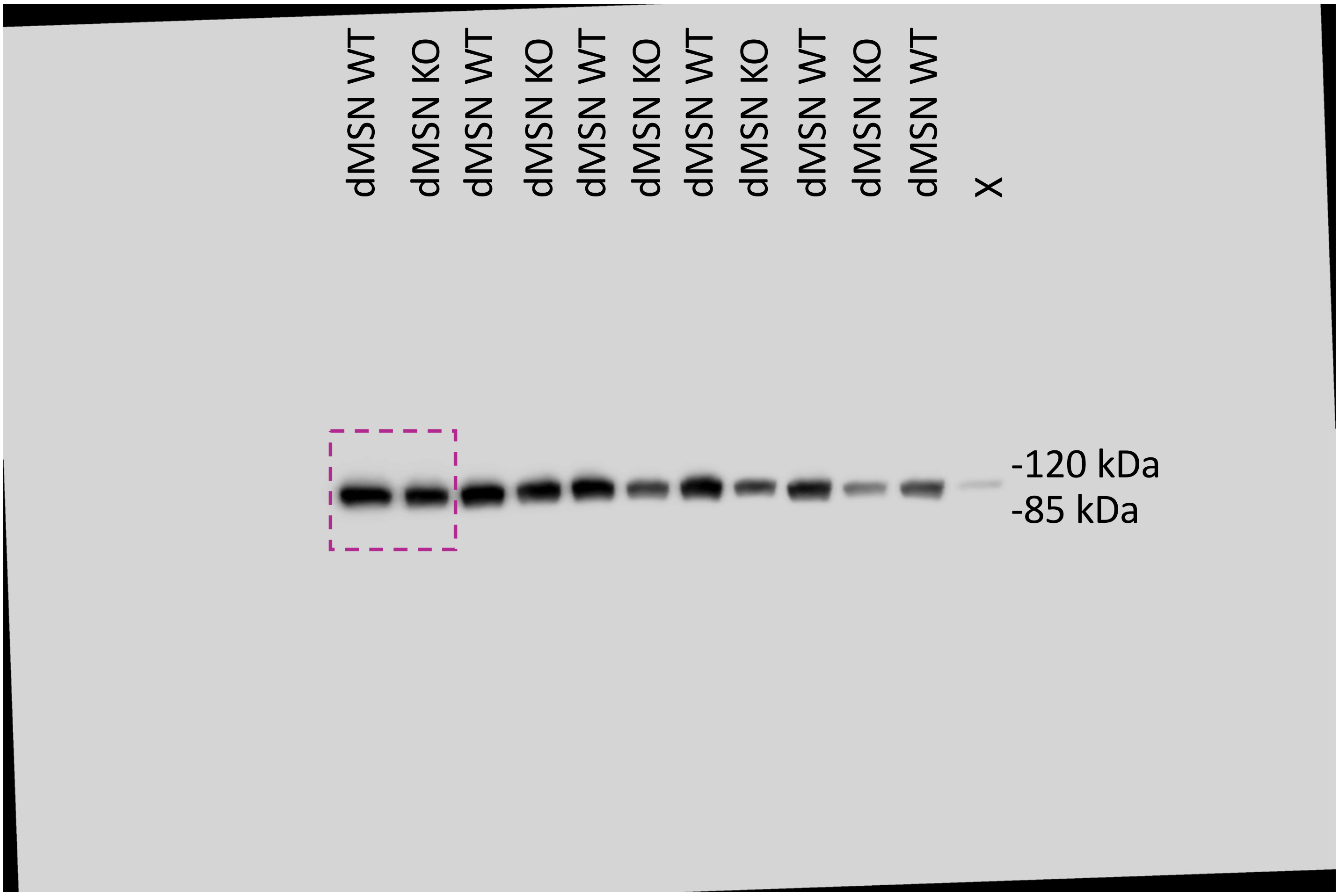

Figure 4B

iMSN KO and iMSN WT
P-Thr34-DARPP32

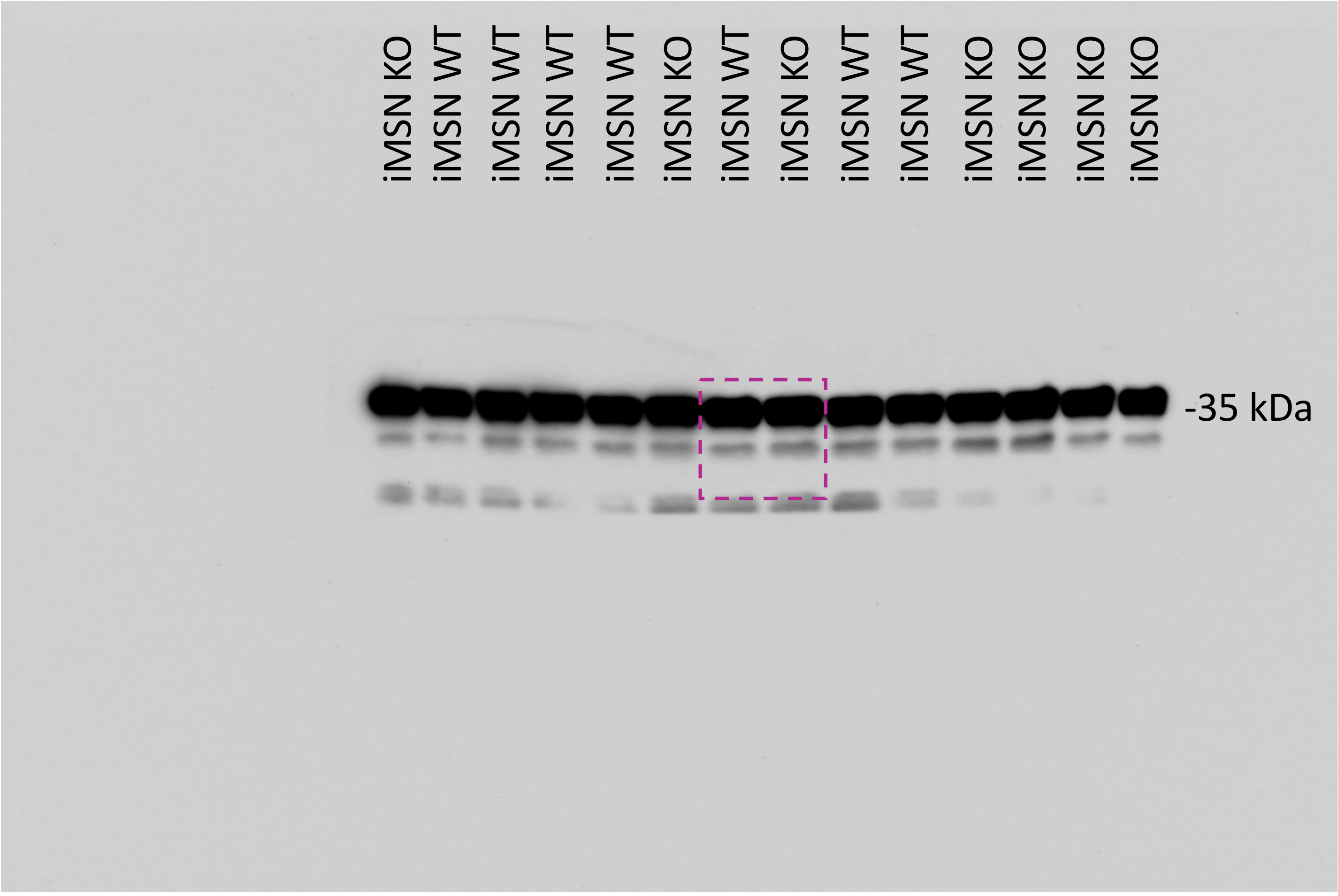

Figure 4B

iMSN KO and iMSN WT
DARPP32

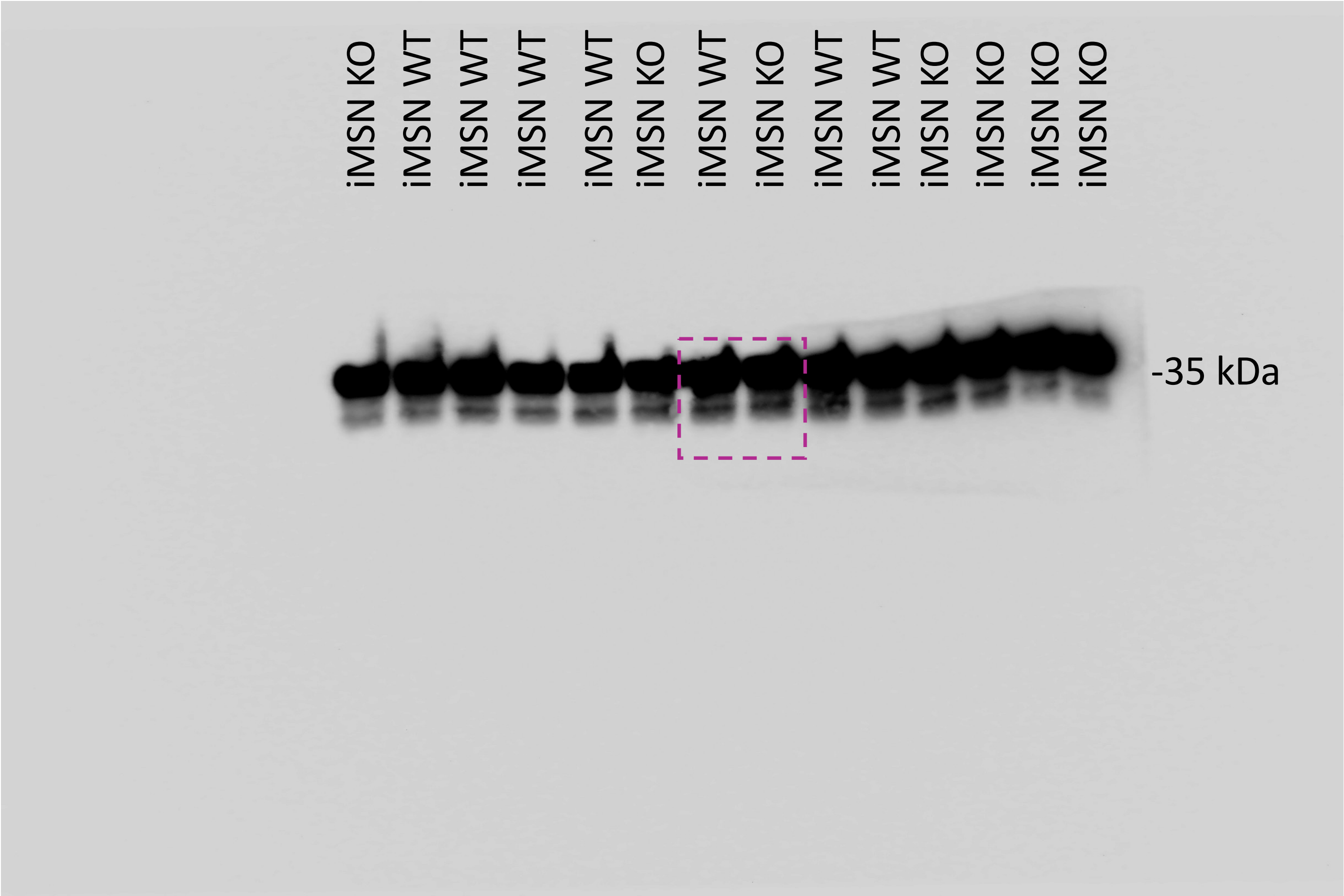

Figure 4B

iMSN KO and iMSN WT
P-Ser845-GluA1

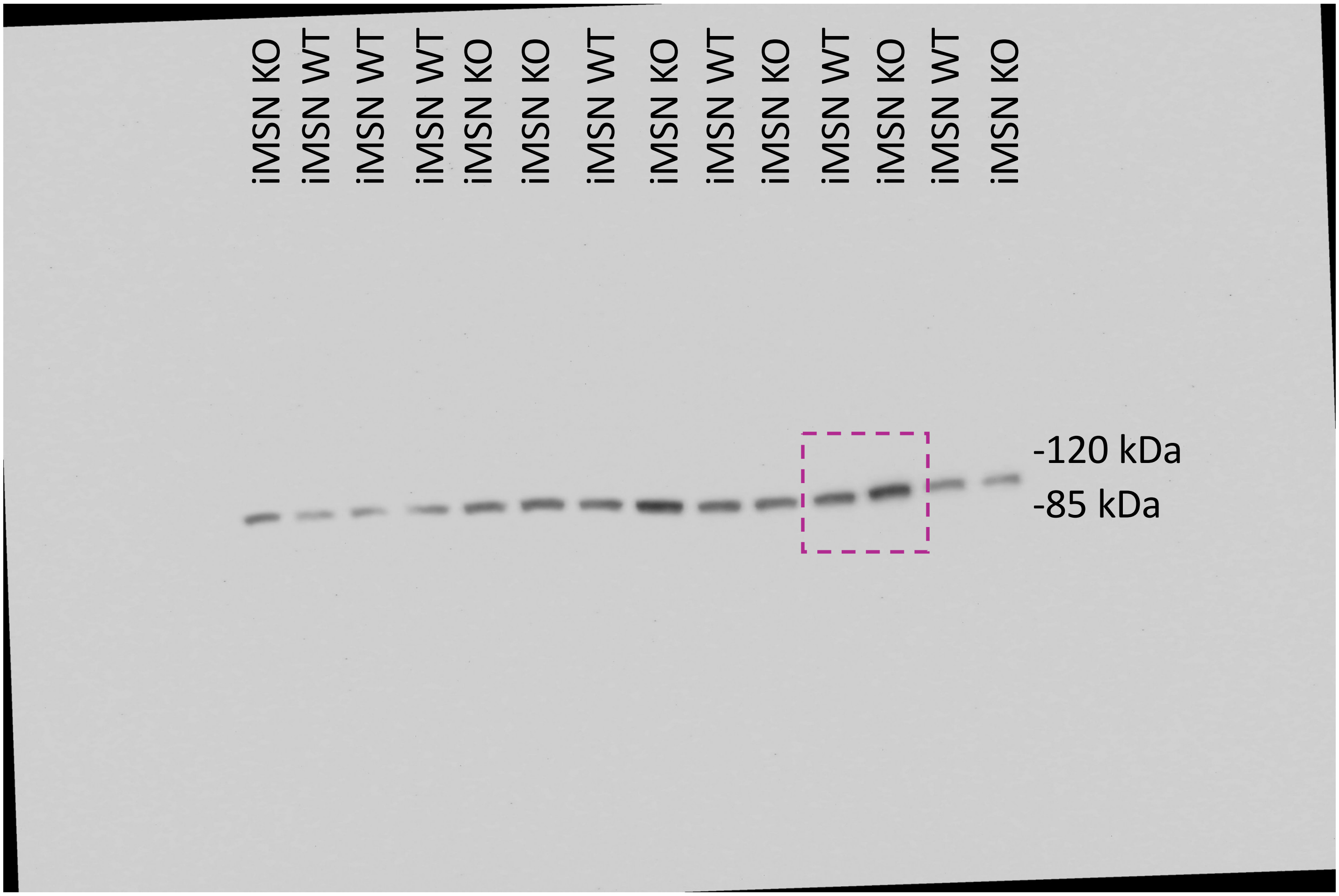

Figure 4B

iMSN KO and iMSN WT
GluA1

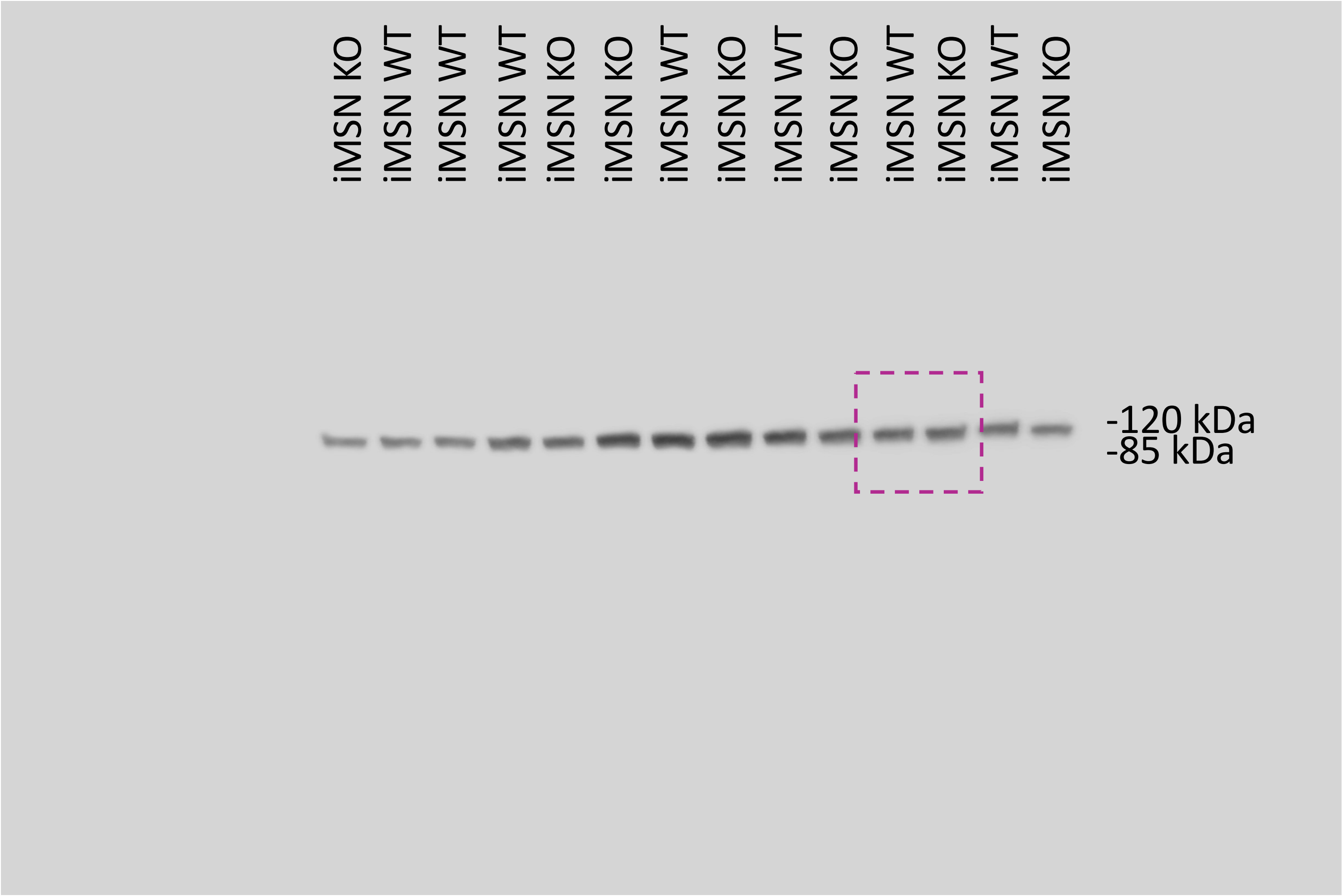

Supplement: S1 Raw Images — (PDF) [file pbio.3003117.s008.pdf]
